# A Novel Image Inpainting Approach for Geometric Calibration of Low-Cost Handheld Thermal Cameras

**DOI:** 10.3390/s26196118

**Published:** 2026-09-27

**Authors:** Mahmod Sahal, Yusuf Abubakar, Kang Li, Nigel Copner

**Affiliations:** 1 Faculty of Computing, Engineering & Science, University of South Wales, Pontypridd CF37 1DL, UK; 2 Higher Institute for Engineering Technologies, Zliten, Libya; 3 Electrical Engineering Department, National College for Nuclear (NCfN) Southern Hub, Bridgwater TA5 2LS, UK; 4 Engineering Unit, Faculty of Business & Physical Sciences, Aberystwyth University, Penglais, Aberystwyth SY23 3FL, UK

**Keywords:** geometric calibration, low-cost thermal camera, image inpainting, firmware overlay, calibration target, emissivity contrast, infrared thermography

## Abstract

This paper presents an integrated calibration framework for consumer thermal cameras whose firmware-embedded display overlays cause failure of conventional corner detection algorithms. The approach combines Navier–Stokes image inpainting for automated overlay removal with UV-printed emissivity contrast targets fabricated on aluminium composite substrates. Under controlled masking of overlay-free imagery, restoration recovers 98.4% of checkerboard corners to within 0.048 px of their ground truth positions, while corners falling within the restored region carry a mean localisation error of 3.34 px and are identified for exclusion. Applied to a consumer handheld thermal camera, the framework raises calibration feasibility from complete failure without preprocessing to successful calibration in every configuration tested, achieving mean reprojection errors of 0.155–0.238 pixels, with held-out errors of 0.328–0.538 pixels on images not used in estimation, with both falling within the range reported in the literature for professional-grade systems. Bootstrap resampling establishes the repeatability of the estimated intrinsics, and hold-out validation confirms that the fitted model generalises to unseen views. Robustness is assessed across two substrate materials, three target scales and both indoor and outdoor environments, comprising 387 images. The complete preprocessing pipeline runs below 50 ms per image. This work demonstrates that computational preprocessing can overcome a hardware-imposed constraint previously assumed to preclude calibration of this class of device.

## 1. Introduction

### 1.1. Background and Motivation

Geometric calibration of thermal cameras establishes the mathematical relationship between image coordinates and real-world geometry, providing the foundation for quantitative measurement, three-dimensional reconstruction, and multi-sensor data fusion [1]. Accurate calibration is essential for transforming thermal imagery from a qualitative visualisation tool into a precise metrological instrument capable of supporting spatial analysis and geometric inference [2].

Infrared thermography (IRT) is widely applied in building diagnostics, process monitoring, and energy auditing [3,4]. Beyond these established uses, thermal imaging increasingly supports quantitative geometric analysis, enabling three-dimensional thermal reconstruction, structural assessment, and defect localisation [5]. Applications now extend to UAV-based infrastructure inspection, medical thermography [6], architectural conservation, cultural heritage documentation, and multi-modal imaging systems [7], where thermal data must be geometrically registered with other sensing modalities. However, realising these capabilities depends critically on reliable geometric calibration that can accurately map thermal measurements to their corresponding spatial locations.

Although infrared imaging technologies have advanced substantially, geometric calibration of thermal cameras remains challenging [8]. Professional-grade systems offer high spatial resolution, stable radiometric behaviour, and sophisticated software support, allowing sub-pixel reprojection errors to be achieved under controlled conditions [8]. These systems nonetheless remain prohibitively expensive for many small laboratories and field-based applications. By contrast, handheld thermal cameras such as those produced by Dongguan Xintai Instrument Co., Ltd. (marketed under the HTI brand, Dongguan, China) and Seek Thermal are available for a few hundred pounds, with infrared resolutions up to 320 × 240 pixels, and they have expanded access to thermography considerably. Despite their accessibility, such cameras exhibit reduced spatial resolution, limited emissivity contrast, and minimal user configurability, which collectively constrain calibration accuracy and repeatability [8].

As thermal sensors are increasingly integrated into consumer devices, unmanned aerial systems, and portable inspection tools, the demand for calibration methodologies that are both accurate and practically deployable has intensified [9]. Bridging the gap between research-grade calibration accuracy and consumer-grade practicality is, therefore, essential if low-cost thermal cameras are to be reliably employed in quantitative measurement, large-scale inspection, and multi-modal reconstruction tasks.

### 1.2. Firmware-Embedded Display Overlays

Consumer handheld thermal cameras render a set of graphical elements into the displayed image. On the device examined in this study, these comprise a central-point temperature cursor, its associated temperature value, highest- and lowest-temperature cursors, a maximum and minimum temperature bar, and status indicators for emissivity, battery state, wireless connectivity, and time.

Not all of these elements are fixed. The manufacturer’s documentation specifies that the highest- and lowest-temperature cursors may be switched off through the Spot setting, and that the maximum and minimum temperature bar may be hidden from the display [10]. Both options were disabled throughout the present work.

The central-point cursor, however, is not configurable. No menu option, key combination, or wireless setting is provided for suppressing it, and it was verified experimentally that the cursor persists in saved images with all available display options disabled. Because it is registered to the geometric centre of the frame, it cannot be avoided by repositioning the camera: a calibration target must occupy the central image region across the range of poses required for well-conditioned parameter estimation, so the cursor necessarily overlaps the pattern in a substantial proportion of acquisitions. The cursor serves as the reference point at which the displayed temperature is measured and is, therefore, integral to the instrument’s primary function [10]. Professional thermal imaging systems typically allow measurement markers to be repositioned or suppressed through configuration software; the devices considered here provide no such facility, and the cursor remains present in every exported image.

The artefact also cannot be circumvented at the export stage. The device records images exclusively in JPG format [10], an eight-bit representation in which the overlay has already been composited into the pixel data, and no radiometric or raw sensor output is documented or available. Two transfer routes exist, mass-storage access over Micro-USB and a wireless interface providing a live stream and image download, and both deliver the same composited representation as the on-device display. The manufacturer provides no software development kit or application programming interface through which sensor-level data might be accessed.

The radiation incident on the microbolometer array is, therefore, measured independently of the overlay, but no user-accessible pathway exists by which the resulting image can be obtained free of it. Geometric calibration of this class of device must consequently operate on overlay-affected image domain data.

The consequence is more severe than a loss of information at the affected pixels. As reported in Section 4.1, applying a standard checkerboard detector to uncorrected imagery does not produce a detection failure but a false detection: the detector converges on the cursor itself, returning a compact grid of points spanning approximately thirty pixels at the frame centre in place of the calibration pattern. Because the algorithm reports success, the resulting correspondences propagate silently into parameter estimation, yielding intrinsics that appear plausible but bear no relation to the physical target. Removal of the artefact prior to feature detection is, therefore, a precondition for calibration rather than an accuracy refinement.

Geometric calibration requires correspondence between known points on the target and their observed positions in the image, established by a feature detector operating on the exported file. Image inpainting is applied for the single purpose of removing the structure that the detector mistakes for the target so that valid correspondences can be obtained; it is a precondition for calibration rather than a component of the calibration model.

### 1.3. Challenges in Low-Cost Thermal Camera Calibration

Beyond the overlay problem described above, low-cost handheld thermal cameras present several further challenges for geometric calibration that stem from fundamental sensor design limitations [8]. Reduced detector resolution limits the precision with which corner positions can be localised, and firmware upsampling of the delivered image does not recover information absent from the underlying measurement. Emissivity-dependent contrast means that the visibility of a calibration pattern depends on material properties and thermal state rather than on illumination, so a target that is clearly resolved under one condition may be indistinct under another.

Calibration target design constitutes a further challenge. Conventional visible light checkerboards exhibit little emissivity variation between printed and unprinted regions, yielding weak feature contrast in the long-wave infrared spectrum. Active targets incorporating resistive heaters or thermoelectric elements can enhance contrast but introduce additional complexity, power requirements, and safety considerations [8]. Passive emissivity-based approaches offer a simpler alternative yet often rely on controlled conditions or higher-grade sensors to achieve consistent performance [11].

As a result of these combined limitations, calibration of handheld thermal cameras commonly yields higher reprojection errors and reduced repeatability compared with professional systems, particularly in field environments. Existing approaches have addressed contrast and localisation, but the artefact described in Section 1.2 presents a different kind of obstacle, one that can prevent calibration entirely rather than merely degrade its accuracy. Section 1.4 reviews the relevant literature.

### 1.4. Related Work

Geometric calibration of thermal cameras underpins quantitative thermography, three-dimensional reconstruction, and multi-sensor fusion [12]. Calibration aims to estimate intrinsic parameters, lens-distortion coefficients, and camera pose that relate thermal imagery to world geometry [1,13]. Compared with visible spectrum systems, thermal cameras are affected by lower spatial resolution, emissivity-dependent contrast, and higher noise levels, which complicate feature localisation and parameter estimation [14].

Existing approaches may be broadly categorised as target-based or targetless. Target-based methods dominate owing to the instability of natural thermal features. Passive planar targets, such as checkerboards or circular grids, have achieved mean reprojection errors of approximately 0.05–0.3 pixels under laboratory conditions [8]. Targetless approaches based on structure from motion or thermal edge tracking simplify data acquisition [15] but remain sensitive to temperature drift and noise, limiting their suitability for portable systems [16].

Recent studies increasingly adopt hybrid optical–digital calibration strategies, combining emissivity contrast target design with algorithmic feature enhancement. Mask-based methods, coded patterns, and iterative corner refinement techniques improve localisation accuracy through computational preprocessing [15,17,18]. These techniques enhance repeatability but assume imagery in which the calibration features are present, even if poorly resolved.

Within the target-based paradigm, thermal visibility and emissivity contrast remain central challenges. Conventional printed checkerboards exhibit near-uniform emissivity, yielding weak feature contrast in long-wave infrared imagery [8,19]. Active targets employing heaters, Peltier modules, or cooling systems generate strong contrast but require power, control electronics, and careful environmental management [17].

Passive emissivity contrast designs offer reduced operational complexity by exploiting material differences, such as polished metals combined with coated regions or low-cost aluminium-based boards [11]. Alternative approaches to physical target design have also been explored. Vidas et al. [15] proposed a mask-based method using geometric cutouts held against thermally contrasting backgrounds, reporting a 78% improvement in mean reprojection error over heated chessboards through enhanced corner visibility. While effective for professional thermal cameras under controlled conditions, such hardware-centric approaches, whether involving heated patterns, printed targets, or physical masks, share a common assumption: that the camera delivers imagery free of sensor-imposed artefacts. To the best of our knowledge, no prior work addresses the case in which the artefact is written into the exported image by firmware and cannot be removed at source.

Recent developments suggest a shift towards computational preprocessing to overcome intrinsic thermal camera limitations. Zhao and Cao [17] demonstrated that external infrared radiation combined with adaptive histogram equalisation and Sauvola binarisation improves corner detection accuracy in low-contrast thermal images, achieving calibration precision comparable to higher-grade systems while reducing hardware complexity. Their work illustrates that algorithmic intervention can compensate for sensor constraints. The present study extends that principle to a different class of problem: rather than enhancing features that are present but poorly resolved, it removes an artefact that overwrites them and induces a false detection.

Despite substantial progress, two limitations persist. First, many reported calibration targets are relatively small, restricting working distance and applicability to large-scale inspection scenarios. Second, approaches relying on active emissivity modulation or external illumination increase system complexity, counteracting the goal of lightweight, field-deployable calibration.

### 1.5. Contributions

This study proposes an integrated geometric calibration framework for low-cost handheld thermal cameras, combining a passively constructed calibration target with computational preprocessing. The work makes three contributions.

First, it identifies and characterises the failure mode by which firmware-embedded overlays prevent geometric calibration. We show that the standard checkerboard detector does not merely fail on affected imagery but returns a false detection on the overlay itself, and we document the absence of any export route by which overlay-free imagery might be obtained from the device.

Second, it introduces a pre-calibration image-conditioning stage that removes the artefact and restores pattern detectability. The stage is validated against ground truth by a controlled masking experiment, in which an overlay of matched geometry is composited over calibration features whose true positions are known, and is compared against alternative reconstruction methods including simple region filling and three inpainting formulations.

Third, it describes a low-cost calibration target of passive construction, fabricated by printing a chessboard pattern in ultraviolet-cured ink on an aluminium composite substrate, and characterises the resulting emissivity contrast by direct measurement.

To the best of our knowledge, this is the first application of image inpainting to the removal of firmware-embedded overlays for thermal camera calibration. The contribution is one of problem identification and system integration rather than algorithm development: the reconstruction method employed is a standard technique, and the novelty lies in establishing that a hardware-imposed constraint previously assumed to preclude calibration of this device class can be resolved through preprocessing alone. Figure 1 summarises the challenges, existing approaches, and the proposed solution addressed in this study.

The scope of the present work is the geometric calibration itself. The applications identified above, which are 3D thermal reconstruction, multi-sensor registration, and quantitative field measurement, depend on accurate intrinsic parameters, and the contribution here is to make those parameters obtainable for a class of devices for which they were previously unavailable. Demonstration of a complete downstream reconstruction pipeline lies beyond the scope of this paper and is identified as future work.

### 1.6. Paper Structure

The remainder of this paper is organised as follows. Section 2 presents the camera calibration model and evaluation metrics. Section 3 describes the camera, calibration targets, emissivity characterisation, acquisition procedure, and the inpainting-based preprocessing pipeline. Section 4 reports the characterisation of overlay-induced detection failure, a controlled validation of geometric preservation, restoration performance across datasets, a comparison of reconstruction methods, and geometric calibration results. Section 5 discusses the findings in relation to existing work and practical deployment. Section 6 concludes.

## 2. Camera Calibration

Geometric camera calibration aims to estimate the intrinsic parameters of the imaging system together with its pose relative to a known reference coordinate system. This process defines how a three-dimensional (3D) point in the world coordinate system is mapped onto a two-dimensional (2D) image plane and constitutes the foundation of metric measurement, three-dimensional reconstruction, and sensor fusion applications [20].

The relationship between world and image coordinates is commonly modelled using the pinhole camera model as illustrated in Figure 2, which assumes an ideal central projection through a single optical centre. Let a 3D point in the world coordinate system be represented in homogeneous form as

(1) $$P_{w}=[X_{w},Y_{w},Z_{w},1{]}^{T}$$

Its corresponding image projection in pixel coordinates,

(2) $$p=[u,v,1{]}^{T}$$

can be expressed as

(3) $$s\left[\begin{array}{c} u\\ v\\ 1 \end{array}\right]=K\left[\begin{array}{cc} R & t \end{array}\right]\left[\begin{array}{c} X_{w}\\ Y_{w}\\ Z_{w}\\ 1 \end{array}\right]$$

where s is a projective scale factor, R and t denote the rotation matrix and translation vector relating to the world and camera coordinate systems, and K is the intrinsic camera matrix.

The intrinsic matrix is defined as

(4) $$K=\left[\begin{array}{ccc} f_{x} & 0 & c_{x}\\ 0 & f_{y} & c_{y}\\ 0 & 0 & 1 \end{array}\right]$$

where $f_{x}$ and $f_{y}$ are the effective focal lengths expressed in pixel units along the horizontal and vertical image axes, and $\left(c_{x},c_{y}\right)$ represents the principal point. This formulation provides a compact description of the internal geometry of the imaging system.

### 2.1. Lens Distortion Modelling

Real imaging systems deviate from the ideal pinhole model due to optical distortions arising from lens geometry, manufacturing tolerances, and sensor alignment. These effects are particularly relevant in compact optical systems, including those employed in low-cost thermal cameras. Lens distortion is commonly modelled using radial and tangential components [21,22].

Let $\left(x,y\right)$ denote the undistorted normalised image coordinates and $\left(x_{d},y_{d}\right)$ the corresponding distorted coordinates. The Brown–Conrady distortion model is expressed as

(5) $$x_{d}=x\left(1+k_{1}r^{2}+k_{2}r^{4}+k_{3}r^{6}\right)+2p_{1}xy+p_{2}\left(r^{2}+2x^{2}\right)$$

(6) $$y_{d}=y\left(1+k_{1}r^{2}+k_{2}r^{4}+k_{3}r^{6}\right)+p_{1}\left(r^{2}+2y^{2}\right)+2p_{2}xy$$

where $r^{2}=x^{2}+y^{2}$ and $k_{1},k_{2},k_{3}$ are radial distortion coefficients, and $p_{1},p_{2}$ are tangential distortion coefficients. These parameters are estimated simultaneously with the intrinsic and extrinsic parameters during calibration to ensure geometric consistency between observed image features and the assumed projection model. In practice, not all coefficients need be estimated; the subset retained should be justified by the resulting fit, as discussed in Section 3.6.

### 2.2. Planar Calibration Procedure

Calibration is typically performed by acquiring multiple images of a planar calibration target observed at different orientations and positions relative to the camera. Planar calibration methods are widely adopted due to their simplicity and robustness, particularly for field-deployable systems [22].

For each calibration image, two-dimensional image points corresponding to known points on the calibration plane are detected and associated with their three-dimensional object coordinates defined in the target reference frame. These correspondences allow an initial estimate of the intrinsic and extrinsic parameters to be obtained. The parameter set is subsequently refined using non-linear optimisation, commonly based on the Levenberg–Marquardt algorithm, to minimise the reprojection error across all observed views.

This formulation is independent of sensor modality and applies equally to visible-spectrum and thermal infrared cameras, provided that reliable feature correspondences can be established between the target and the image data.

### 2.3. Accuracy Metrics

Calibration accuracy is evaluated by comparing the detected image coordinates of calibration features with their reprojected positions computed from the estimated camera parameters. For an observed point i, the reprojection error is defined as

(7) $$e_{i}=\sqrt{\left(u_{i}-{\hat{u}}_{i}{)}^{2}+(v_{i}-{\hat{v}}_{i}{)}^{2}\right.}\;$$

where $\left(u_{i},v_{i}\right)\;$ are the detected image coordinates and $\left({\hat{u}}_{i},{\hat{v}}_{i}\right)\;$ are the corresponding reprojected coordinates obtained from the calibrated model.

Two commonly reported global measures are the mean reprojection error (MRE),

(8) $$\mathrm{MRE}=\frac{1}{N}\sum_{i=1}^{N}e_{i}$$

and the root mean square Error (RMSE),

(9) $$\mathrm{RMSE}=\sqrt{\frac{1}{N}\sum_{i=1}^{N}e_{i}^{2}}$$

where N is the total number of detected points across all calibration images. Sub-pixel reprojection errors are generally interpreted as indicative of high-quality geometric calibration.

### 2.4. Considerations for Thermal Camera Calibration

Although the mathematical framework of camera calibration is invariant with respect to spectral range, several factors specific to long-wave infrared (LWIR) imaging influence practical calibration performance. Thermal cameras typically exhibit lower spatial resolution and higher noise levels than visible light systems, increasing sensitivity to feature localisation errors. In addition, LWIR optics may experience temperature-dependent variations in refractive index, which can affect the stability of intrinsic parameters under changing environmental conditions [14].

These characteristics place greater emphasis on robust feature detection and stable calibration targets when applying standard geometric calibration models to thermal imagery. Consequently, practical thermal camera calibration frameworks must account for both optical and sensor-related effects to ensure that the underlying projection model can be reliably estimated.

## 3. Materials and Methods

### 3.1. Thermal Imaging System

All experiments were conducted using an HT-A9 handheld thermal infrared camera (Dongguan Xintai Instrument Co., Ltd., Dongguan, Guangdong, China (HTI brand)). The device employs an uncooled microbolometer operating in the long-wave infrared (LWIR) spectral range of 8–14 μm. It provides a native detector resolution of 320 × 240 pixels, with recorded images internally upsampled to 640 × 480 pixels. The camera field of view is 56° × 42°, and the camera operates at a frame rate of 9 Hz. A representative view and key specifications of the thermal camera used in this study are shown in Figure 3 and Table 1.

This device was selected as representative of low-cost, consumer-grade thermal imagers increasingly used in field applications, where calibration capabilities remain limited. A defining characteristic of this class of devices is the presence of firmware-embedded graphical overlays, including a central temperature cursor and textual annotations. These overlays are permanently fused into the recorded thermal images and cannot be disabled through software configuration, as discussed in Section 1.2. Consequently, they introduce high-contrast, non-physical features that interfere with feature detection during geometric calibration. As illustrated in Figure 4, these overlays occlude the calibration pattern and hinder reliable corner detection.

#### Verification of Overlay Persistence

The claims made in Section 1.2 regarding overlay persistence were verified experimentally before data acquisition. The highest- and lowest-temperature cursors were disabled through the Spot setting, and the maximum and minimum temperature bar was hidden, following the manufacturer’s documentation [10]. Images were then captured and inspected to confirm that these elements were absent and that the central-point cursor and its temperature annotation remained.

Each available export route was tested in turn, and the resulting files examined for format, bit depth, and overlay content. The wireless interface was accessed at the address given in the manufacturer’s documentation and the delivered stream compared to the on-device display. The results are summarised in Table 2.

All available routes deliver an eight-bit JPG representation in which the central-point cursor is already composited into the pixel data. No radiometric or raw output was obtainable by any route, and no software development kit or application programming interface was provided by the manufacturer. The overlay could not, therefore, be removed at source, and the pre-calibration conditioning stage described in Section 3.5 was developed in response.

### 3.2. Calibration Target Design and Fabrication

Planar checkerboard calibration targets were fabricated using Dibond^®^ aluminium composite panels (3A Composites GmbH, Singen, Germany), selected for their high dimensional stability, low thermal deformation, and structural rigidity. Each panel comprises two 0.3 mm aluminium skins bonded to a 2 mm polyethylene core, forming a lightweight yet mechanically robust substrate suitable for repeated experimental use.

To evaluate scale robustness and accommodate varying imaging distances, three target sizes were produced: a compact 230 × 210 mm target for short-range acquisition, an A3 format (420 × 297 mm) for intermediate distances, and an A2 format (594 × 420 mm) serving as the primary calibration target.

Two surface types were investigated to assess material-dependent calibration performance. The primary targets were fabricated from silver Dibond^®^, featuring an RAL 9006 metallic-effect coated surface representative of industrial materials. All three target sizes were produced using this material, with checkerboard patterns printed on the metallic face.

In addition, a validation target was fabricated using white Dibond^®^, which exhibits higher emissivity and reduced reflectivity compared to the metallic variant. This target was used to assess the generalisability of the calibration framework to materials with differing thermal properties. A single A2-sized target was produced for this purpose, with the pattern printed on the glossy white surface. Emissivity characterisation for both substrate materials is detailed in Section 3.3.

Checkerboard patterns were applied using an industrial UV flatbed printing process (HP Acuity Press; HP Inc., Palo Alto, CA, USA) with commercial-grade UV-curable ink (Fujifilm Uvijet KN; Fujifilm Speciality Ink Systems Ltd., Broadstairs, UK). This process enables high-resolution deposition (nominally 1200 × 1200 dpi) and produces well-defined geometric boundaries with consistent surface properties. Importantly, the use of commercially available printing technology ensures that the fabrication process remains accessible and reproducible without requiring specialised materials or equipment.

Pattern geometry was selected to balance calibration accuracy, manufacturability, and experimental flexibility. The compact 230 × 210 mm target employed 25 mm squares in a 7 × 8 configuration (6 × 7 internal corners), enabling reliable detection at short distances. For the larger A2 and A3 targets, 50 mm squares were used to maintain visibility at increased working distances. The A3 target adopted a 7 × 5 configuration (6 × 4 internal corners), while the A2 target employed a denser 10 × 7 configuration (9 × 6 internal corners) to provide improved geometric constraint during calibration. This two-scale design strategy simplifies fabrication while ensuring sufficient feature coverage across a 2.4× size range. Calibration targets used in this study across different sizes and materials are shown in Figure 5. Key specifications for each target configuration are summarised in Table 3.

### 3.3. Emissivity Characterisation

The emissivity of the materials used in the calibration targets was determined experimentally under controlled indoor conditions using a reference-based thermographic method. The target comprised a checkerboard pattern printed with UV-curable black ink on an aluminium composite substrate. A strip of matte black electrical tape (assumed emissivity $\varepsilon_{ref}\approx 0.95$) was applied across both a UV-printed region and an unprinted region of the RAL 9006-coated substrate, following the established thermographic practice [11,23] as shown in Figure 6.

To account for environmental reflections, the apparent reflected temperature $T_{ref}$ was measured using a crumpled aluminium foil positioned at the target location and observed from the same viewing geometry as the thermal camera, consistent with ISO 18434-1 recommendations and established thermographic methodologies [24]. The reflected temperature was 17.0 °C and was constant during each measurement sequence.

Following uniform heating of the target, three temperature quantities were recorded at each of three temperature levels spanning approximately 38–50 °C: the reference tape temperature $T_{tape}$, the apparent temperature of the test surface $T_{meas}$, and the reflected temperature $T_{ref}$.

The emissivity of the test surface was estimated relative to the reference tape using:

(10) $$\varepsilon =\varepsilon_{ref}\cdot \frac{T_{meas}-T_{ref}}{T_{tape}-T_{ref}}$$

where $\varepsilon_{ref}$ denotes the emissivity of the reference tape.

This formulation assumes approximate thermal equilibrium across the target surface, negligible conductive heat transfer between adjacent materials during measurement, and a linear relationship between apparent temperature and emitted radiance over the narrow temperature interval considered. Individual measurements and the resulting emissivity estimates are given in Table 4.

The UV-cured ink and the RAL 9006-coated substrate differ in emissivity by Δε = 0.17. The substrate value is consistent with a pigmented coating rather than bare metal; the underlying aluminium is not exposed at the imaged surface.

Because the derived values depend on two assumed reference quantities, their sensitivity to those assumptions was examined. Table 5 reports the emissivities obtained when the reference-tape emissivity is varied across 0.93–0.97 and when the reflected temperature is varied by ±2 °C.

Varying the assumed reference tape emissivity shifts the derived values by at most 0.036, and the contrast between them by 0.007. Varying the reflected temperature by ±2 °C shifts them by at most 0.039, and the contrast by 0.025. The emissivity contrast is, therefore, substantially more robust than either individual value, since errors in the reference quantities affect both surfaces in the same direction and largely cancel in the difference.

The measured values were consistent across repeated trials and fall within ranges reported for comparable materials in the literature [11]. The experimentally determined emissivity values characterise the target surfaces and were used to inform the radiometric configuration of the thermal camera during data acquisition. They are not presented as evidence that the measured contrast alone is sufficient for corner detection; that question is considered in Section 5.4.

### 3.4. Data Acquisition

Thermal image datasets were acquired under both indoor and outdoor conditions to capture a range of environmental and radiometric scenarios. Indoor experiments were conducted at ambient temperatures of 22–25 °C under controlled conditions, while outdoor acquisitions were performed under natural illumination and variable thermal backgrounds.

Calibration targets were mounted vertically, and the camera was fixed on a tripod to ensure geometric stability. Acquisition distances ranged from approximately 0.8 m to 2 m, depending on target size. For each dataset, images were captured with systematic variation in orientation and viewing angle, approximately ±20° to ±40°, ensuring sufficient geometric diversity for reliable calibration. Image fusion between the infrared and visible sensors was disabled throughout, so that all recorded imagery derives from the thermal detector alone. The emissivity settings used during acquisition were selected on the basis of the material properties reported in Section 3.3.

All datasets were acquired following thermal preconditioning. The target was warmed using a portable electric fan heater, which was then removed, and images were captured from the freely cooling target once a stable thermal state was reached; the heater was neither present nor operating during acquisition. Preconditioning was repeated between acquisition sequences as the target returned toward ambient. Indoor target temperatures ranged from approximately 20 to 50 °C and outdoor from 10 to 40 °C, with the lower outdoor range reflecting increased convective and radiative loss.

The target itself is passive in construction: it contains no embedded heating elements, thermoelectric modules, control electronics, or power supply and requires no external illumination. Thermal conditioning is an acquisition-stage procedure using standard equipment rather than the property of the target.

Thermal images were recorded using multiple colour palettes, primarily white-hot and black-hot, to evaluate the robustness of the preprocessing pipeline under different visual representations. Although these palettes do not alter the underlying radiometric measurements, they influence apparent contrast and the visibility of both the calibration pattern and the embedded overlay. In particular, the overlay renders lighter than the surrounding scene under one palette and darker under the other, so the two palettes exhibit different behaviour in the mask detection stage described in Section 3.5. For final calibration, the white-hot palette was used throughout. Because the firmware overlay occupies a fixed region of the image, its relative size varies with target scale. This produces differing levels of artefact coverage across datasets, providing a natural test of robustness for the proposed preprocessing framework.

A total of 387 images were processed through the restoration pipeline. Of these, 159 were used across the four calibration runs reported in Section 4.5, selected on the basis of full board visibility and pose diversity, ensuring adequate coverage of orientation and working distance.

### 3.5. Image Inpainting Framework

To enable reliable geometric calibration, a preprocessing stage was introduced to remove firmware-embedded artefacts prior to feature detection. The stage isolates and suppresses the non-physical overlay while preserving the geometrically relevant thermal structure surrounding it. The overall calibration workflow and the detailed conditioning pipeline are illustrated in Figure 7 and Figure 8, respectively. Each thermal image was processed using a Python (version 3.12.4) pipeline implemented with OpenCV (version 4.10.0), comprising artefact detection, binary mask generation, mask refinement, image restoration, and quality assessment.

The overlay renders lighter than the surrounding scene against dark backgrounds and darker against bright ones, depending on the colour palette in use, so a fixed intensity threshold is unreliable. Detection instead exploits three properties that remain invariant: the artefact is rendered by firmware at a fixed position at the frame centre, occupies a predictable spatial extent, and departs sharply from its immediate local surroundings in either direction.

A local median is subtracted within a search region of ±140 px about the frame centre to form a local contrast image. Connected components exceeding 60 grey levels from the local median, in either direction, and those falling within an area range of 40–4000 px are retained as candidate overlay regions. Components lying further than 140 px from the frame centre are rejected. The resulting binary mask is dilated by 3 px to enclose the anti-aliased boundary of the rendered artefact. Where both bright and dark components are present, as occurs when the cursor and its annotation straddle regions of differing scene intensity, the complementary masks are combined by logical union.

Image restoration was performed using the Navier–Stokes method as implemented in OpenCV, invoked as cv2.inpaint (src, mask, inpaintRadius = 7, flags = cv2.INPAINT_NS). This approach models image intensity as the stream function of an incompressible fluid, allowing structural information to propagate into the masked region along isophote directions.

Let $I\left(x,y\right)$ denote the image intensity and Ω the region to be reconstructed. The inpainting process seeks a steady-state solution satisfying:

(11) $$\nabla ⊥I\cdot \nabla \left(\Delta I\right)=0,\;in\;\Omega$$

This is subject to boundary conditions defined on ∂Ω. This formulation ensures that isophotes are smoothly continued into the missing region, preserving geometric continuity at the boundary.

The method was selected in preference to diffusion-based, variational, and exemplar-based alternatives for reasons following from the structure of the artefact. The concealed region is small and structurally simple, containing not texture but the linear light–dark boundaries of a checkerboard pattern. The Navier–Stokes formulation transports structure along isophote directions defined at the mask boundary, continuing pattern edges across the reconstruction rather than blurring them. An exemplar-based method offers no corresponding advantage where there is no texture to reproduce and incurs greater computational cost; simple diffusion would tend to blur the very pattern edges whose continuation is the object of the operation. This reasoning is examined empirically in Section 4.3.3.

Two distinct evaluations were performed. Restoration behaviour across all acquired datasets was characterised by root mean square difference and structural similarity computed between the restored image and the image as delivered by the camera, together with processing time per image. Because no reference exists for the scene concealed by the overlay, these quantities describe the extent and locality of the operation rather than reconstruction accuracy; their interpretation is set out in Section 4.2.

Reconstruction accuracy proper was assessed separately by a controlled experiment in which ground truth is available by construction, described in Section 4.4. There, an overlay of matched geometry is composited over calibration features whose true positions are known, and the displacement of recovered corner positions is measured directly. Root mean square error, peak signal-to-noise ratio, and structural similarity are additionally reported over the masked region only.

### 3.6. Geometric Calibration Procedure

Following preprocessing, geometric calibration was performed using Zhang’s method [22] under a pinhole camera model, implemented in MATLAB R2025b using routines from the Computer Vision Toolbox.

Checkerboard corners were detected using detect Checkerboard Points applied to each image set as a whole rather than to individual images. When supplied with a set, the detector enforces a consistent board geometry across all images and rejects detections inconsistent with it. This is significant for the present application: applied to single images, the detector converges on the firmware cursor and returns a false grid, as reported in Section 4.1. Detected corners were refined to sub-pixel accuracy.

Corresponding three-dimensional object points were defined on a planar grid with spacing determined by the physical square size of the target, 25 mm for the compact configuration and 50 mm for the A2 and A3 configurations. Intrinsic parameters, comprising the focal lengths ($f_{x},f_{y}$) and principal point coordinates ($c_{x},c_{y}$), were estimated by non-linear optimisation. Lens distortion was modelled using two radial coefficients ($k_{1},k_{2}$) based on the Brown–Conrady formulation given in Section 2.1; tangential distortion and skew were not estimated.

A note on pixel units: The detector resolution of the camera is 320 × 240, and images are delivered at 640 × 480 by firmware upsampling. All pixel-denominated quantities reported in this paper are expressed in upsampled image pixels, corresponding to approximately half that value in native detector pixels.

Calibration was performed across four configurations spanning two target scales and both acquisition environments. Accuracy was evaluated using the mean reprojection error defined in Section 2.3. Because mean reprojection error measures internal fit rather than accuracy, two further analyses were performed: bootstrap resampling over random image subsets, to establish the repeatability of the estimated parameters, and hold-out validation, in which parameters estimated from 70 per cent of images were used to predict corner positions in the withheld 30 per cent. Both are reported in Section 4.5.

## 4. Results

Without preprocessing, geometric calibration of the test camera was not achievable: the corner detector converged on the firmware cursor rather than the calibration target, returning correspondences unrelated to the physical pattern. With preprocessing, the calibration pattern was recovered in every image tested, and calibration succeeded in all four configurations examined. The results are presented first for the detection failure that motivates the framework, and then for the primary Silver A2 dataset, followed by validation experiments assessing robustness across target material and scale. Geometric preservation is then quantified against known ground truth, before calibration performance is analysed for the largest (A2) and smallest (230 × 210 mm) calibration targets.

### 4.1. Overlay Interference and Detection Failure

The firmware overlay does not degrade corner detection progressively; it causes the detector to fail categorically, and to do so without indication of error.

Applied to uncorrected imagery, the checkerboard detector consistently converged on the central temperature cursor rather than on the calibration target. Figure 9 illustrates the behaviour. The left panel shows the cursor and its temperature annotation superimposed on the calibration pattern. The middle panel shows the detector output: the pattern origin has been assigned to the cursor itself, the coordinate axes radiate from that point, and the reprojected points cluster within the cursor region rather than following the physical checkerboard. The resulting correspondences bear no relation to the target, yet the detector reports a successful detection. The right panel shows the same frame after restoration, in which the complete grid of internal corners is recovered, and the pattern origin is correctly assigned to a target corner.

This behaviour was observed across the calibration datasets and prevented parameter estimation in every case. In a controlled examination of twenty frames, the detector returned a grid in fifteen; in these, the returned arrangement was a 4 × 4 configuration of 16 points spanning approximately 30 × 30 pixels, centred within one pixel of the frame centre, with a mean point spacing of 7.0 pixels. The calibration target in the same frames presents 54 internal corners spanning several hundred pixels. In the remaining five frames, the detector returned with no result.

The failure mode is more damaging than an absence of detection. Because the algorithm reports success, the resulting correspondences propagate into parameter estimation without any indication of error, yielding intrinsic parameters that appear plausible but describe nothing physical.

Two mechanisms contribute. The cursor obscures the intensity structure at any calibration corner it covers, so no saddle-point response is generated at those locations. Separately, the high-contrast boundaries of the cursor and its annotation generate saddle-like responses that compete with genuine corner candidates during grid fitting. Rendered at the exact geometric centre of the frame, these responses form a compact and internally consistent grid hypothesis which the detector accepts in preference to the true pattern.

An artefact covering under one per cent of the frame is, therefore, sufficient to prevent calibration entirely. Conventional strategies for improving thermal corner detection—contrast enhancement, adaptive thresholding, iterative sub-pixel refinement—operate on features that are present but poorly localised. They cannot recover features that have been overwritten or suppress a false grid that the detector prefers to the true one. Removal of the artefact prior to feature detection is thus a precondition for calibration rather than a refinement of its accuracy, and the following section characterises the restoration stage introduced for that purpose.

### 4.2. Inpainting Performance

#### Primary Calibration Silver A2 Target

The primary dataset consists of 178 thermal images acquired using a Silver A2 UV-printed Dibond^®^ calibration target. Each image contains a firmware-embedded central temperature cursor and associated artefacts, which were automatically detected and removed using the proposed inpainting framework. The quantitative results are summarised in Table 6.

Outdoor acquisitions (=74) yielded a mean structural similarity of 0.9912 ± 0.0005 and a root mean square difference of 135.0 ± 16.2 grey levels, with mask coverage ranging from 0.44 to 0.56 per cent. Indoor acquisitions (*n* = 104) yielded 0.9916 ± 0.0005 and 117.2 ± 20.4, respectively, with coverage from 0.50 to 0.59 per cent. Across all 178 images, structural similarity remained at 0.9914 ± 0.0005. Per-image results and their distributions are shown in Figure 10.

The interpretation of these quantities requires care, because the natural reading of them is not the correct one. No ground truth exists beneath the overlay: the portion of the scene concealed by the cursor was never recorded by the camera, and no reference image of it is obtainable. These measures, therefore, compare the restored image against the overlay-bearing original, and within the masked region a large difference indicates that the overlay has been removed rather than that the underlying structure has been poorly reconstructed. The root mean square difference is accordingly reported here as a measure of removal extent, not of reconstruction accuracy.

The structural similarity index carries a different and more useful meaning. Computed over the whole image, with the masked region covering under one per cent of the frame, it is dominated by the region the stage does not modify. Its value above 0.99, therefore, confirms that restoration is strictly local and leaves the remainder of the frame undisturbed. This is a necessary property of a preprocessing stage placed ahead of a calibration since any change to the peripheral corner positions would corrupt the parameters recovered from them, and it is the property that the index is well suited to demonstrate. It is not evidence of pixel-level reconstruction accuracy within the masked region and is not presented as such. Reconstruction accuracy proper is quantified against known ground truth in Section 4.4.

The correlation between removal extent and mask coverage was weak under outdoor conditions (r = −0.066) and stronger indoors (r = −0.599). These values are reported for completeness; they reverse sign between datasets, and no physical interpretation is placed upon them.

Computational performance was evaluated in terms of per-image processing time. Indoor images were processed in 0.024 s on average, while outdoor images required approximately 0.039 s due to increased thermal complexity. Processing time distributions for both datasets are shown in Figure 11**,** while summary statistics are provided in Table 7.

### 4.3. Validation Experiments

To assess the generalisability of the proposed pre-calibration image inpainting framework, validation experiments were conducted to evaluate robustness with respect to calibration target material and scale. For each validation case, the results are compared against the Silver A2 baseline. Computational performance remained consistent across all target scales and materials, with processing times below 50 ms per image in all cases.

#### 4.3.1. Material Independence

The results for the white A2 calibration target are summarised in Table 8. Compared to the Silver A2 baseline, the RMSE increased by approximately 11.9%, while SSIM remained high (0.9911±0.0008). A stronger negative correlation between the RMSE and mask coverage (r=−0.540, p<0.001) was observed compared to the baseline dataset (r=−0.33), indicating an increased dependence of reconstruction error on artefact size. Despite these variations, reconstruction performance remained consistently above 0.99 across both materials. Representative inpainting results for the A2 white validation target are shown in Figure 12.

#### 4.3.2. Scale Robustness

Scale robustness was evaluated using A3 (297 × 420 mm) and compact (230 × 210 mm) calibration targets, and the results are shown in Table 9.

The A3 dataset (*n* = 38) produced RMSE = 151.1±22.0 and SSIM = 0.9908±0.0003, maintaining high structural similarity while exhibiting increased reconstruction error relative to the Silver A2 baseline. A strong negative correlation (r=−0.807, p<0.001) was observed. Representative inpainting results for the Silver A3 target are shown in Figure 13.

For the compact target, RMSE = 117.1±17.9 and SSIM = 0.9917±0.0006, matching or exceeding baseline performance. A positive correlation (r=+0.409, p<0.001) was observed.

Despite differences in projected target size and relative overlay coverage, reconstruction performance remained consistent across both target scales.

#### 4.3.3. Reconstruction Method and Inpainting Radius

The choice of reconstruction method and of inpainting radius was examined empirically rather than assumed. Ten variants were applied to an identical set of 192 test images, described in Section 4.4, in which an overlay of matched geometry is composited over calibration features whose true positions are known. Each variant, therefore, received the same input and was scored against the same reference. Table 10 shows a comparison of reconstruction methods and inpainting radii. Corner displacement is measured against ground truth as described in Section 4.4. Reported times include image reading and writing, which account for approximately 24 ms of each figure.

Structure-aware reconstruction substantially outperforms simple removal of the artefact. Replacing the masked region with a local mean or median reduces corner displacement only marginally relative to leaving the overlay in place, at 5.69 and 5.61 px against 5.83 px, whereas the inpainting methods reduce it by between 32 and 49 per cent. Removal of the artefact alone is, therefore, not sufficient: the reconstruction must continue the pattern structure across the masked region if corner geometry is to be recovered.

Among the inpainting methods, the biharmonic formulation achieved the lowest displacement at 2.948 px together with the highest masked structural similarity, followed by Telea at 3.537 px and Navier–Stokes at 3.749 px. These differences lie within approximately one standard deviation of one another. Navier–Stokes was retained for its balance of accuracy against computational cost, with the biharmonic method requiring approximately 50 per cent greater processing time for a difference of 0.8 px in a region containing on average fewer than one calibration corner per image.

Reconstruction accuracy was insensitive to the inpainting radius above 5 px, with the mean displacement varying by 0.10 px across radii from 5 to 15 px and masked root mean square error varying by 0.3 grey levels. A radius of 7 px was retained. Displacement of corners outside the restored region remained below 0.07 px for every variant, confirming that the choice of method affects only the reconstructed region and leaves the surrounding pattern undisturbed.

### 4.4. Geometric Validation Against Ground Truth

Restoration preserves the positions of the calibration corners it does not cover to within 0.048 pixels and recovers the pattern reliably enough for detection in every trial. This section establishes those figures against known ground truth.

The measures reported in Section 4.2 and Section 4.3 cannot demonstrate this because the scene concealed by the overlay was never recorded and no pixel-level reference for it exists. A controlled experiment was, therefore, devised in which ground truth is available by construction.

Sixteen frames were selected in which the firmware overlay falls on the background rather than on the calibration target, leaving the pattern unobstructed. Corner positions detected in these frames constitute ground truth. The overlay was then extracted from the same imagery and composited over the calibration pattern at twelve positions per frame, with each chosen to occlude one or more internal corners, giving 192 trials. Because the temperature annotation differs between frames while the cursor itself does not, a bank of extracted overlays was retained and one drawn at random for each placement, reproducing the variability of the real artefact.

The complete pipeline was then applied, including automatic mask detection. The restoration stage was given only the approximate location of the artefact, corresponding to the prior it holds in normal operation where firmware renders the cursor at the frame centre, and it recovered the mask from image evidence alone; it was not supplied with the applied mask. Mean recall of the detected mask against the applied overlay was 0.975. Corners were re-detected in the restored images using the same detector settings as the ground truth pass and matched to their ground truth positions by nearest neighbour.

The calibration pattern was detected in all 192 restored images. Figure 14 illustrates the procedure for a representative trial, in which the composited overlay covered four internal corners. These are marked distinctly in the right-hand panel, while the remaining corners were recovered at their ground truth positions.

Corner localisation displacement was measured for every matched corner across the 192 trials, separating corners that fell within the restored region from those outside it. The results are summarised in Table 11.

Displacement is exactly zero for 95.96% of corners outside the restored region. Restoration modifies only pixels within the mask, so corners elsewhere are localised from bit-identical image data, and the detector returns identical coordinates. Since the proportion at zero exceeds 95%, the 95th percentile is zero; the mean and standard deviation arise from a small number of corners adjacent to the mask boundary.

Corners outside the restored region, comprising 98.4 per cent of all detected corners, were recovered to within 0.048 px of their ground truth positions, with a median displacement of zero. Restoration, therefore, leaves the great majority of calibration features geometrically undisturbed, at a scale well below the reprojection errors reported in Section 4.5.

Corners falling within the restored region carried a mean displacement of 3.338 px. The restoration stage removes the artefact and returns a locally plausible continuation of the pattern but does not recover the true corner positions beneath it. This is an expected consequence of reconstructing from boundary information alone, and it delimits precisely what the preprocessing stage achieves: it restores the detectability of the calibration pattern without disturbing the features that remain visible, rather than recovering the scene content concealed by the overlay. Because these corners are identified during mask detection, they may be excluded from parameter estimation where required. Displacement within the restored region showed no dependence on the proportional extent of the applied overlay across the range examined.

Because true ground truth is available in this controlled setting, the reconstruction measures used in Section 4.2 and Section 4.3 were additionally computed over the masked region only, against the true reference. The results are given in Table 12.

These are the values these measures take when computed against a genuine reference within the reconstructed area, and they differ substantially from the whole-image figures reported in Section 4.2 for the reasons given there. The whole-frame structural similarity of 0.989 in the same trials confirms once again that the operation is confined to the masked region.

### 4.5. Geometric Calibration Results

Geometric calibration was performed to evaluate the effectiveness of the proposed preprocessing framework in enabling accurate intrinsic parameter estimation. Zhang’s method was applied to inpainted thermal images across multiple target configurations and colour palettes, as described in Section 3.6. Representative examples of inpainted thermal images used for calibration are shown in Figure 15 and Figure 16, demonstrating consistent removal of firmware artefacts while preserving the checkerboard geometry required for reliable corner detection. Calibration accuracy was evaluated using the mean reprojection error (MRE). Sub-quarter-pixel accuracy was achieved across all configurations, with the lowest error observed for the smallest target (230 × 210 mm). Indoor calibration using the Silver A2 target gave a mean reprojection error of 0.191 pixels and outdoor calibration of 0.219 pixels, as demonstrated in Section 4.5.2. Intrinsic parameters estimated from the A2 target were consistent between environments; estimates from the compact target differed between sessions, as examined in Section 4.5.2.

#### 4.5.1. Effect of Inpainting

The impact of the proposed preprocessing stage on calibration feasibility was evaluated by comparing calibration outcomes with and without inpainting. Without preprocessing, calibration failed in all cases (0% success), as firmware-embedded overlays prevented reliable detection of checkerboard corners (see Section 3.1). In contrast, applying the proposed inpainting framework resulted in successful calibration for all datasets (100% success rate), as summarised in Table 13.

The A2 calibration target enabled stable calibration across working distances of approximately 0.8–2 m, as illustrated in Figure 17, which presents representative views across this range.

#### 4.5.2. Repeatability and Hold-Out Validation

Because calibration accuracy depends on which images are selected and on the range of camera poses they span, the repeatability of the estimated parameters was assessed by bootstrap resampling. For each configuration, 200 random subsets of 20 images were drawn from the available set and calibrated independently. Results at a subset size of 15 were statistically indistinguishable and are not reported separately. Table 14 shows repeatability of estimated intrinsic parameters. Values are bootstrap means ± standard deviation over 200 random subsets of 20 images; 95 per cent confidence intervals in brackets.

Parameters estimated from the A2 target were consistent between acquisition environments, with the focal length differing by 0.10 px between indoor and outdoor datasets with confidence intervals overlapping substantially. Estimates from the compact target differed between sessions, at 681.79 and 693.07 px, with the first radial coefficient likewise differing beyond the bootstrap intervals. This reflects the weaker geometric constraint that a smaller target subtending a smaller image area places on focal length and indicates that the larger target should be preferred where parameter accuracy rather than portability is the priority.

Mean reprojection error measures how closely the fitted model reproduces the observations used to estimate it and is, therefore, a measure of internal fit rather than of accuracy. To assess whether the estimated parameters generalise, a hold-out validation was performed in which parameters estimated from 70 per cent of the images in each set were used to predict corner positions in the remaining 30 per cent. Table 15 shows hold-out validation. Parameters are estimated from 70 per cent of images and are used to predict corner positions in the withheld 30 per cent.

Held-out reprojection errors between 0.328 and 0.538 px, with ratios to training error between 1.67 and 2.26, indicate that the fitted model predicts corner positions in unseen views to a comparable order of accuracy and does not simply absorb pose-specific error. Reprojection error is, therefore, a meaningful indicator of calibration quality for these datasets rather than an artefact of overfitting.

#### 4.5.3. Automatic Exclusion of Occluded Corners

Section 4.4 established that corners recovered from within the restored region carry a mean localisation error of 3.34 px. Because these corners are identified during mask detection, they can be excluded automatically from parameter estimation.

The mask produced by the detection stage of Section 3.5 is retained and used to classify detected corners. Following corner detection on the restored image, any corner whose refined coordinates fall within the mask is excluded from parameter estimation for that image, while remaining available in images where it is not occluded. The test requires no additional information since the mask is already computed as part of the restoration stage and is applied automatically.

Applied to the Silver A2 indoor dataset, exclusion removed 1.97% of detected corners, a mean of 1.07 per image, retaining a mean of 52.9 of 54. At least one corner was affected in 73.8% of images. Mean reprojection error fell from 0.1926 px to 0.1860 px, and the estimated focal length differed by 0.28 px, an order of magnitude below the bootstrap confidence interval reported in Section 4.5.2. The step is, therefore, implementable automatically and marginally improves the fit, while the calibration remains robust to its omission.

## 5. Discussion

The results demonstrate that firmware-embedded overlays constitute a fundamental barrier to the geometric calibration of consumer thermal cameras. Unlike conventional limitations, such as sensor noise or low thermal contrast, which degrade calibration accuracy progressively, these overlays introduce dominant non-physical features that cause the corner detector to fail categorically, converging on the artefact rather than on the calibration target. The proposed pre-calibration conditioning framework addresses this limitation entirely in the image domain, enabling calibration without modification to the camera hardware or to the underlying calibration model.

### 5.1. Inpainting Performance and Robustness

The restoration stage operated successfully across all four target configurations and both acquisition environments, producing imagery from which the calibration pattern was detected in every case. Three aspects of this result merit discussion: what the reconstruction measures establish, what the controlled validation adds, and how performance varies with target material and scale.

What the reconstruction measures establish. As set out in Section 4.2, the absence of any ground truth reference beneath the overlay means that the difference measures computed across the acquired datasets cannot speak to reconstruction accuracy. This is a consequence of the artefact itself: the overlay is composited into the image by the camera firmware before the file is written, so the concealed scene content was never recorded in any form. The measures reported, therefore, establish two narrower propositions. First, that the overlay is removed, evidenced by the correspondence between removal extent and mask coverage across datasets. Second, that removal is confined to the masked region and leaves the surrounding image undisturbed, evidenced by structural similarity above 0.99 throughout. The second of these is not incidental: a preprocessing stage that perturbed peripheral corner positions would corrupt the calibration it precedes, and demonstrating strict locality is a precondition for placing any such stage ahead of parameter estimation.

What the controlled validation adds. The experiment reported in Section 4.4 answers the question the reconstruction measures cannot. Restoration recovers 98.4 per cent of calibration corners to within 0.048 px of their true positions and enables detection in every frame, but it does not accurately reconstruct the corners it covers, which carry a mean displacement of 3.34 px. This distinction matters for how the method should be understood and applied: the preprocessing stage restores the detectability of the calibration pattern, rather than recovering the scene content concealed by the overlay. The distinction is not merely semantic. Because the affected corners are identified during mask detection, they may be excluded from parameter estimation where required, retaining the great majority recovered to sub-tenth-pixel accuracy. Without restoration, no corners are usable at all since the detector converges on the cursor and returns a false grid.

The method comparison of Section 4.3.3 further establishes that removal alone is insufficient. Replacing the masked region with a local mean or median leaves corner displacement close to that obtained with no restoration at all, whereas structure-aware reconstruction reduces it by a third or more. The operative mechanism is, therefore, the continuation of pattern structure across the masked region, not the suppression of the artefact as such. This finding supports the choice of an isophote-transporting formulation over simpler alternatives and indicates that the reconstruction stage is doing geometric work rather than merely erasing.

Variation with material and scale. Because the overlay occupies a fixed region of the frame irrespective of the target imaged, its proportional coverage of the calibration pattern varies with target scale, and the datasets consequently present the stage with mask coverages spanning 0.31 to 0.97 per cent. Performance was maintained across this range: structural similarity remained above 0.99 in every dataset, the pattern was detected in all 387 images, and no configuration required adjustment of the detection or restoration parameters, which were held fixed throughout. The white A2 target showed a greater spread in removal extent, at ±38.8 against ±21.2 grey levels for the silver baseline, consistent with its wider range of mask coverage; the central tendency was comparable. The within-dataset correlations between removal extent and mask coverage are negative in three datasets and positive in the fourth, while the ordering across datasets is positive throughout. A relationship that reverses sign between the within-group and between-group views cannot be explained by a single mechanism, and no physical interpretation is placed upon these values.

Processing times below 50 ms per image indicate that the stage imposes negligible overhead on a calibration workflow and would remain tractable if applied to image sequences rather than to individual calibration frames.

### 5.2. Calibration Target Performance

The measured emissivity contrast between printed and unprinted regions (Δε = 0.17) is a property of the target surfaces, as characterised in Section 3.3. Because all datasets were acquired following thermal preconditioning, the contribution of this contrast to pattern detectability cannot be separated from that of the imposed thermal gradient; this is addressed in Section 5.4.

The comparison between silver and white substrates indicates that the framework is not restricted to a single material. The white target exhibited slightly higher reconstruction error and greater sensitivity to mask coverage but retained sufficient structural fidelity for the pattern to be detected reliably. This demonstrates that the framework is not restricted to a specific material but performs optimally when passive contrast is maximised.

Importantly, the use of commercially available UV flatbed printing aligns with fabrication approaches reported in prior work. Usamentiaga et al. [11] utilised UV-curable pigment inks with industrial flatbed printers, while ElSheikh et al. [8] employed UV-curable inks on metallic substrates. The present study demonstrates that comparable performance can be achieved using widely accessible commercial printing services at a cost of approximately £30–45 per A2-sized target, supporting the reproducibility and scalability of the proposed target design.

### 5.3. Calibration Performance

The geometric calibration results establish the functional role of the preprocessing framework. Without conditioning, the detector converges on the firmware cursor and calibration cannot proceed. With conditioning, calibration succeeded in every configuration tested, achieving mean reprojection errors between 0.155 and 0.238 px. This transition demonstrates that overlay removal is not an enhancement step but a prerequisite for calibration in this class of device.

Bootstrap resampling establishes that the estimated parameters are repeatable across image subsets rather than dependent on a particular selection, and hold-out validation confirms that they predict corner positions in unseen views to a comparable order of accuracy. The latter is significant because mean reprojection error measures how closely the fitted model reproduces the observations used to estimate it, and a model that absorbed pose-specific error would show a substantially larger error on withheld images. Ratios between 1.67 and 2.26 indicate that this is not the case.

Target configuration was found to influence calibration accuracy. The compact target yielded the lowest reprojection error, which can be attributed to favourable feature scaling and stronger local thermal gradients within the image, although the bootstrap intervals reported in Section 4.5.2 show that it constrains focal length less tightly than the larger target. The A2 target, while not achieving the lowest reprojection error, produced the most repeatable parameter estimates and enabled calibration across extended working distances of approximately 0.8–2 m, demonstrating its suitability for field applications. Parameters estimated from the A2 target were consistent between indoor and outdoor acquisition, whereas estimates from the compact target differed between sessions, reflecting the weaker geometric constraint imposed by a smaller target rather than any property of the preprocessing stage. These observations indicate that calibration performance is governed by the interaction between thermal contrast, feature scale, and imaging conditions and that the larger target should be preferred where parameter accuracy rather than portability is the priority.

Once this constraint is addressed, the reprojection errors obtained (0.155–0.238 pixels) fall within the range commonly reported for professional thermal camera systems [9,13,19]. Table 16 provides a comparative overview of representative calibration approaches, including differences in camera specification, target design, fabrication method, and reported accuracy. This comparison is indicative rather than controlled: the studies differ in detector resolution, acquisition protocol, and error definition, and the reported values are not directly equivalent. In particular, the errors reported here are expressed in upsampled image pixels, corresponding to approximately half that value in native detector pixels, whereas the comparison systems report errors at their native resolution. Within these constraints, the results indicate that following preprocessing, a consumer thermal camera can achieve geometric calibration accuracy of the same order as that reported for higher-grade systems.

From the perspective of existing calibration approaches, the proposed method complements and extends established strategies. Passive emissivity-based methods [8,11] have demonstrated strong performance with artefact-free imagery; the present work confirms this principle while extending it to consumer cameras with overlay-corrupted images through computational preprocessing. Earlier hardware-centric approaches, such as the mask-based method of Vidas et al. [15], demonstrated the potential of target design innovation but remained constrained by the requirement for thermal background generation and artefact-free camera output. Active approaches based on thermal excitation achieve strong contrast but require controlled conditions and additional hardware [25,26]. By contrast, the passive approach adopted here enables immediate deployment under ambient conditions.

More broadly, the results support an emerging shift towards computationally assisted calibration, as introduced in Section 1.3. Recent work by Zhao and Cao [17] demonstrates that adaptive preprocessing can compensate for low contrast in thermal images, improving feature detectability without specialised hardware. The present work addresses a distinct but complementary challenge: the removal of calibration-blocking overlays. Unlike contrast limitations, which degrade accuracy gradually, firmware overlays prevent calibration entirely. The convergence of these independent lines of work from physical target design [15] through passive target design [11] to image enhancement and computational preprocessing [17]; this work suggests that algorithmic intervention may offer a scalable path to expanding access to calibrated thermal imaging.

In practical terms, the proposed framework comprising a consumer thermal camera (~£400) and a passively fabricated calibration target (~£30–45) enables quantitative thermal imaging for educational, field inspection, and multi-camera deployment scenarios where the cost of professional systems would be prohibitive.

### 5.4. Limitations and Future Directions

All experiments used a single camera model. The central-point temperature cursor is not, however, a peculiarity of this device: it serves a common function across handheld thermal cameras, indicating the point at which the displayed temperature is measured, and it is rendered at the geometric centre of the frame for that reason. The proposed framework operates on the exported image and makes no assumption about how the artefact is generated, requiring only that it occupy a bounded region of predictable position and extent. It is, therefore, expected to transfer to other devices exhibiting comparable overlay behaviour. This expectation has not been tested, however, and the overlay geometry, rendering polarity, and available display settings differ between manufacturers. The quantitative results reported here are accordingly stated for the HT-A9, and confirmation across additional camera models is identified as future work.

All calibration datasets were acquired following thermal preconditioning. The contribution of emissivity contrast to pattern detectability, therefore, cannot be separated from that of the imposed thermal gradient. The emissivity values reported in Section 3.3 characterise the target surfaces but do not establish that the measured contrast alone would be sufficient for corner detection at thermal equilibrium. Establishing this would require a controlled acquisition under ambient conditions without conditioning, which is identified as future work.

Corners recovered from within the restored region carry a localisation error of approximately 3.3 px and should be treated as unreliable. Although these corners are identifiable during mask detection and may, therefore, be excluded from parameter estimation, the framework does not recover the scene content beneath the artefact, and no method operating on the exported image alone could do so.

The distortion model retained two radial coefficients; the contribution of higher-order radial and tangential terms was not examined systematically. The comparison with published systems in Section 5.3 is indicative rather than controlled. The study was also restricted to planar checkerboard targets, and performance on alternative calibration geometries such as circular patterns or coded markers remains to be investigated.

Future work may extend the framework to dynamic overlay detection, real-time video inpainting, stereo and multi-camera configurations in which thermal imagery must be registered with other modalities, alternative target geometries, such as circular grids or coded markers, and confirmation of transferability across additional camera models.

## 6. Conclusions

Firmware-embedded display overlays prevent geometric calibration of a class of low-cost handheld thermal cameras, not by degrading corner localisation but by causing the standard checkerboard detector to return a false detection on the overlay itself. Because the affected devices export only eight-bit composited imagery and provide no radiometric output or software interface, the artefact cannot be avoided at source.

This work has shown that the obstacle can be removed computationally. A preprocessing stage combining automatic mask detection with Navier–Stokes inpainting restored pattern detectability in all imagery tested. Controlled masking against known ground truth established that it recovers 98.4 per cent of calibration corners to within 0.048 px of their true positions, while the remaining 1.6 per cent, which fall within the reconstructed region, carry a larger localisation error and are identified for exclusion. Comparison against alternative restoration methods confirmed that structure-aware reconstruction is required: simple removal of the artefact reduces corner displacement only marginally.

Calibration of a consumer thermal camera using a UV-printed target fabricated on Dibond^®^ aluminium composite achieved mean reprojection errors of 0.155–0.238 px across four configurations spanning two target scales and both indoor and outdoor environments. Bootstrap resampling established the repeatability of the estimated intrinsics, and hold-out validation confirmed that they generalise to unseen views. The complete restoration pipeline runs below 50 ms per image.

The target is fabricated using commercial UV flatbed printing at approximately £30–45 per A2 panel and contains no embedded heating elements, thermoelectric modules, or control electronics. Combined with a consumer thermal camera at approximately £400, the system enables quantitative thermal imaging at a fraction of the cost of professional equipment.

To the best of our knowledge, this is the first application of image inpainting to the removal of firmware-embedded overlays in thermal camera calibration. The contribution is one of problem identification and system integration rather than algorithm development: the reconstruction method employed is a standard technique, and the significance lies in establishing that a hardware-imposed constraint previously assumed to preclude calibration of this device class can be resolved through preprocessing alone.

These results are established for the HT-A9 and devices with comparable firmware overlay behaviour; generalisation across consumer thermal cameras requires testing of additional models.

The progression from hardware-centric calibration solutions as proposed by Vidas et al. [15] through computational preprocessing for contrast enhancement by Zhao and Cao [17], to the removal of calibration-blocking artefacts demonstrated here, suggests that algorithmic approaches offer a scalable path to expanding access to calibrated thermal imaging, particularly for devices whose hardware constraints cannot be modified by the user.

## Figures and Tables

**Figure 1:**
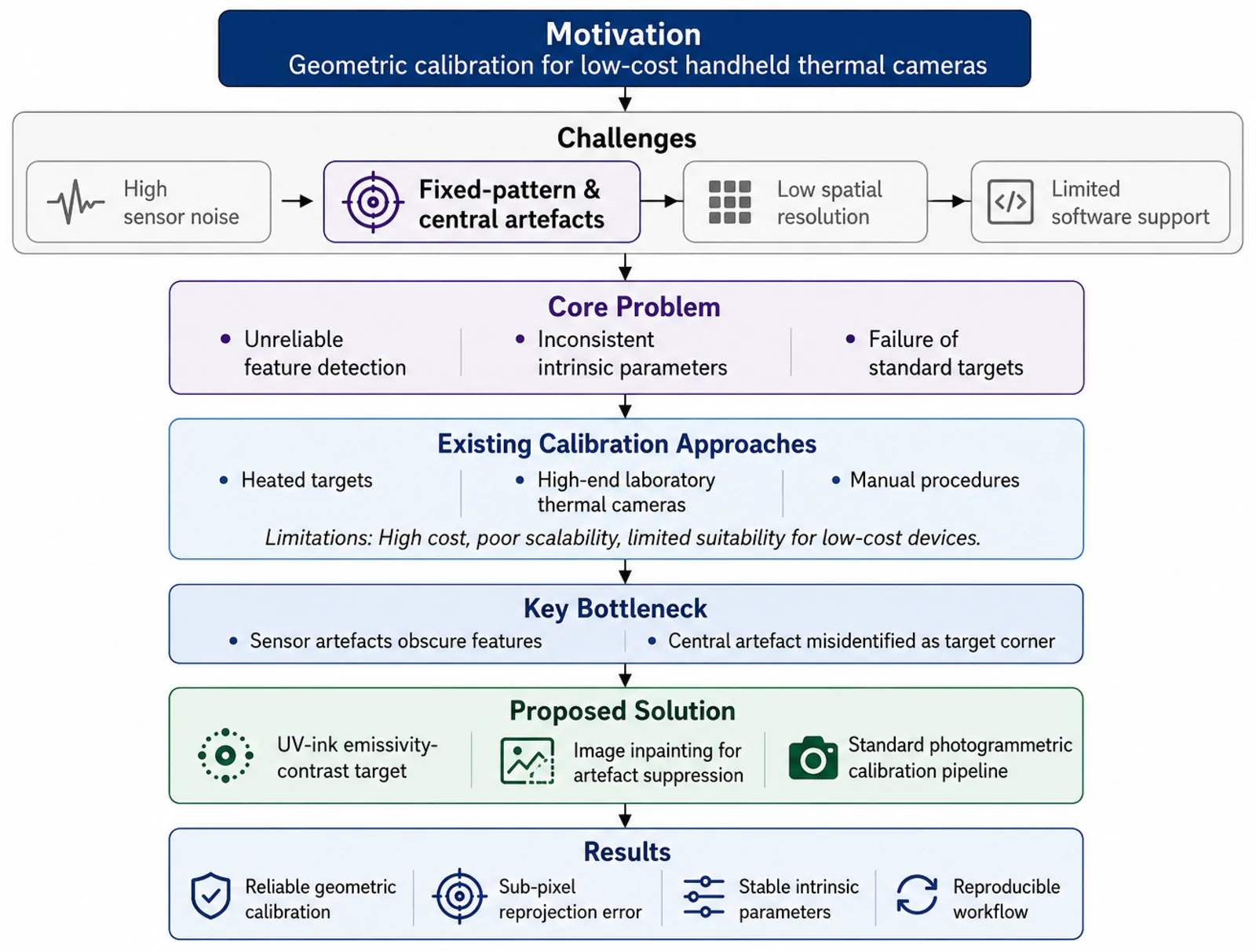
Research framework: from calibration challenges in low-cost thermal cameras to the proposed solution.

**Figure 2:**
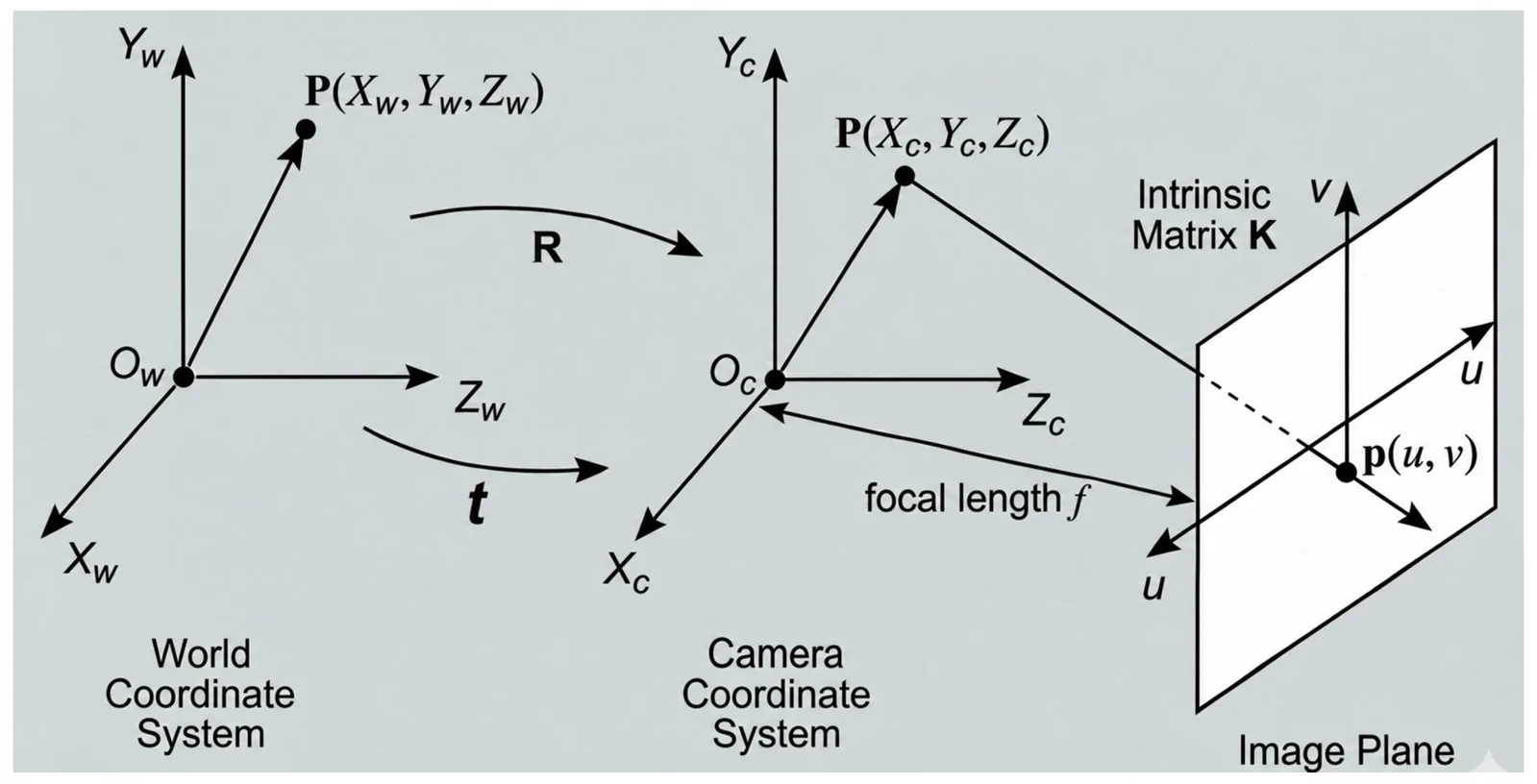
Pinhole camera model showing the transformation from world coordinates to image coordinates via rotation **R**, translation **t**, and intrinsic matrix **K**.

**Figure 3:**
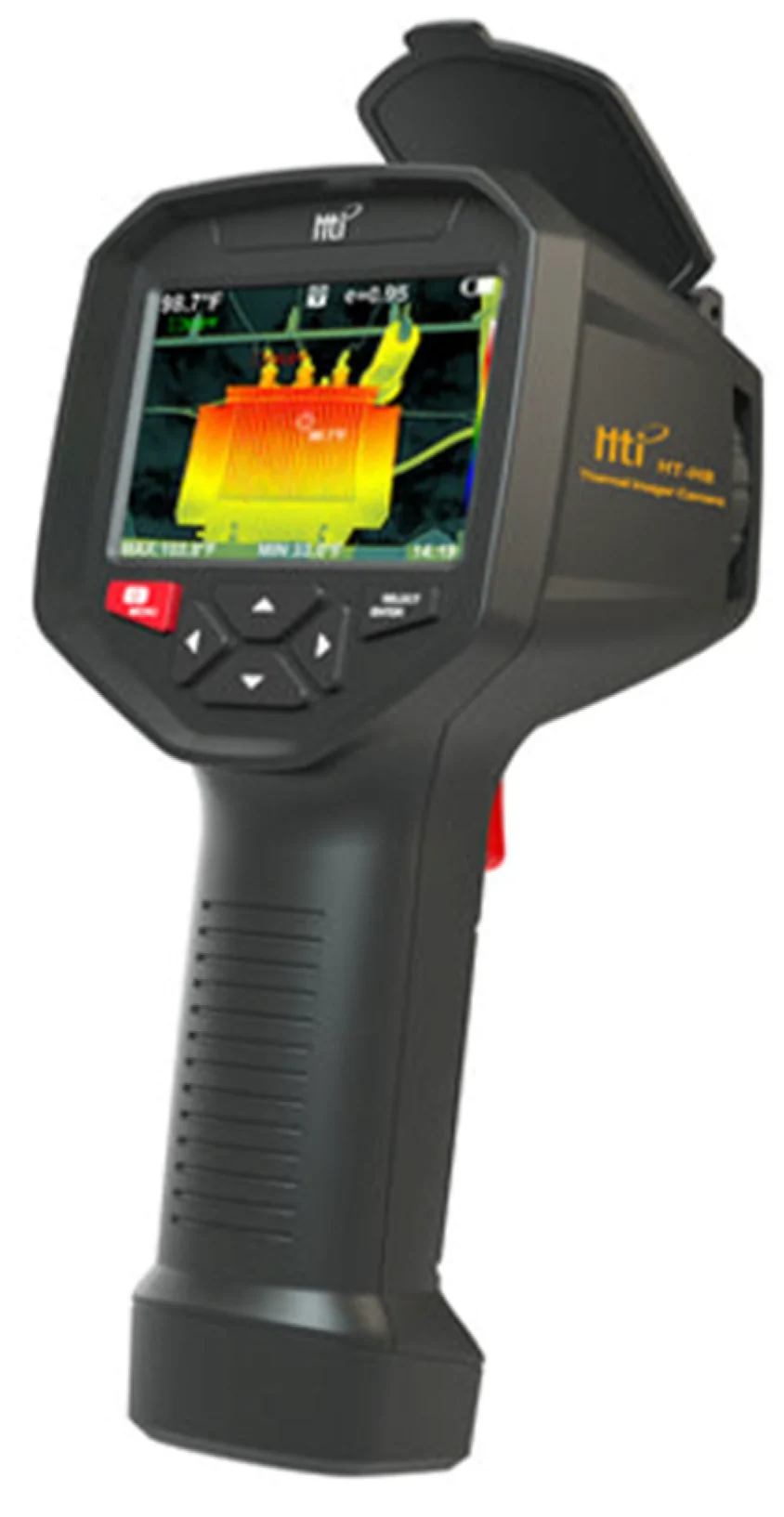
A representative view of the HT-A9 thermal camera.

**Figure 4:**
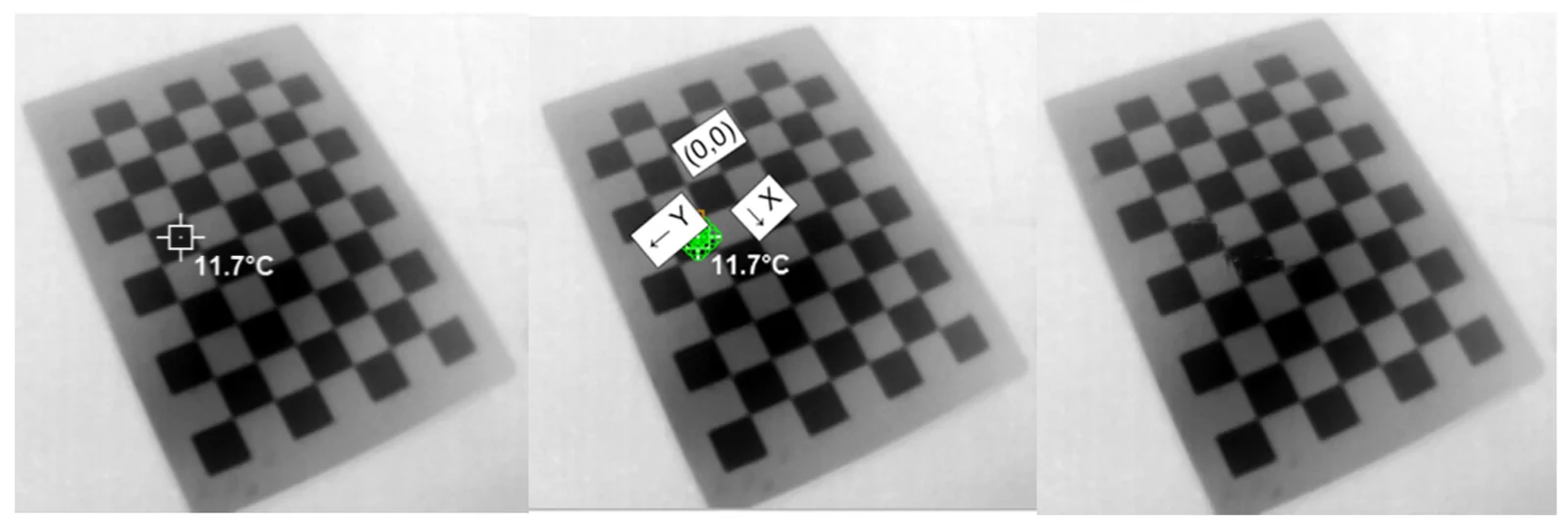
Firmware-embedded overlay interference on the HT-A9 thermal camera. (**Left**): central temperature cursor. (**Middle**): coordinate axes and cursor overlay during calibration. (**Right**): image after inpainting-based overlay removal.

**Figure 5:**
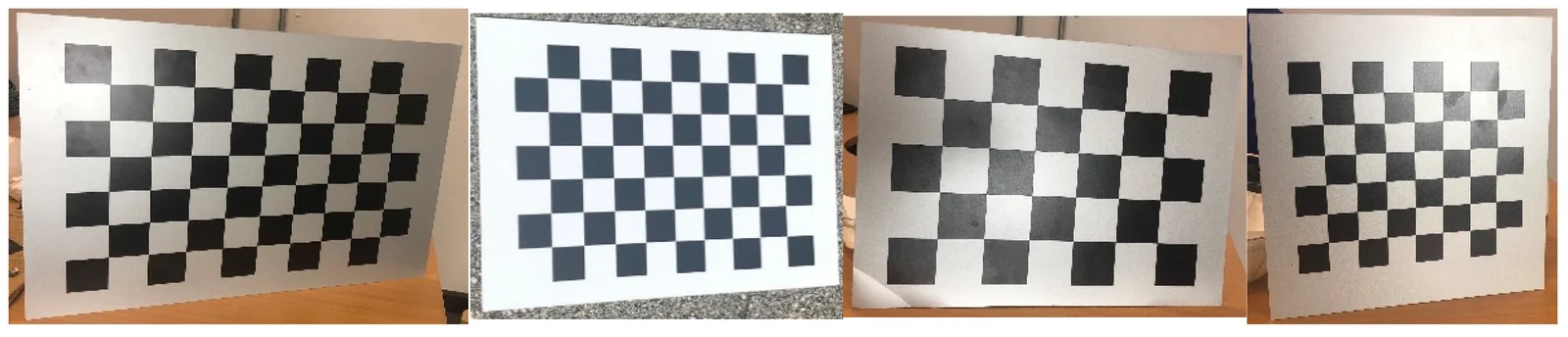
Calibration targets fabricated for this study. (**Left**): Silver A2 (594 × 420 mm). (**Middle**): white A2 and Silver A3 (420 × 297 mm). (**Right**): silver (230 × 210 mm).

**Figure 6:**
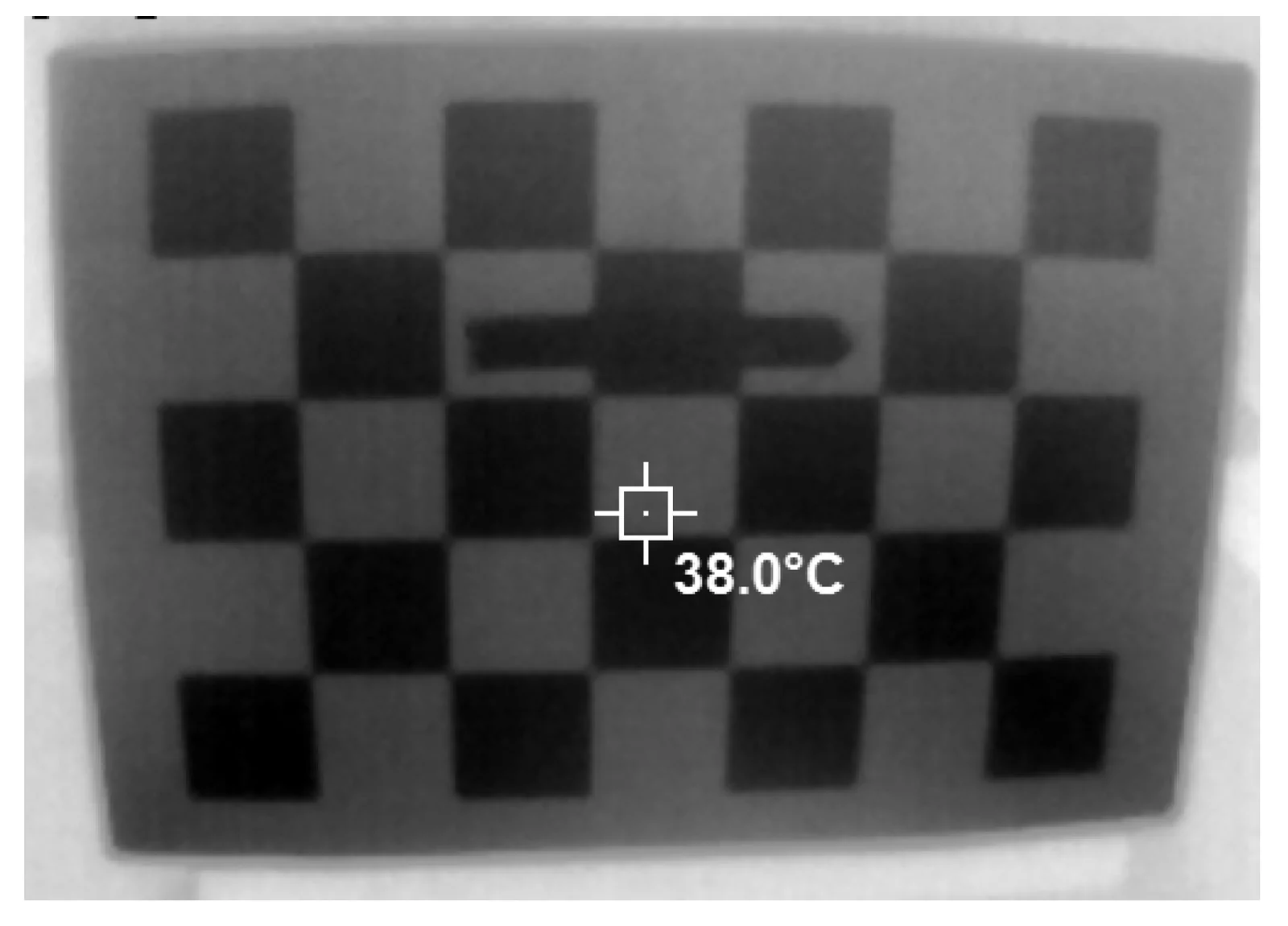
Emissivity measurement setup: matte black electrical tape applied across UV-printed and RAL 9006-coated aluminium regions of the calibration target.

**Figure 7:**
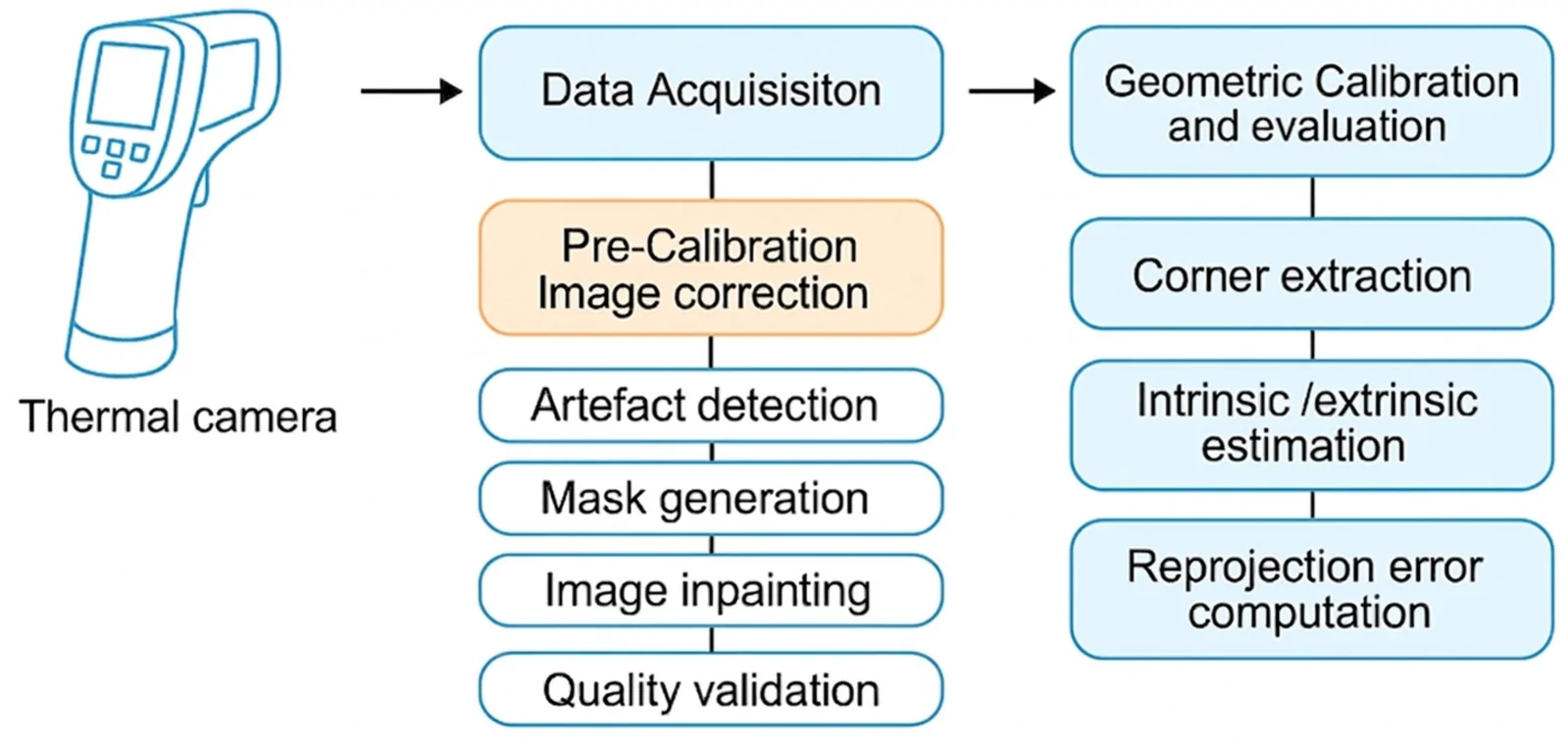
Overall calibration workflow: from thermal image acquisition through pre-calibration image correction to geometric calibration and evaluation.

**Figure 8:**
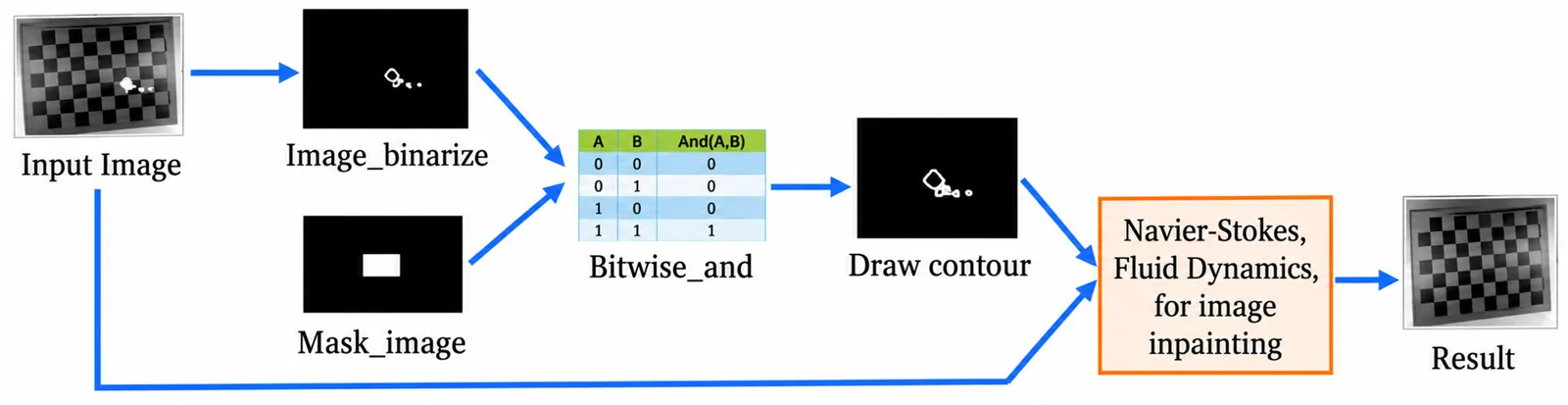
Detailed inpainting pipeline: artefact detection, mask generation, and image inpainting.

**Figure 9:**
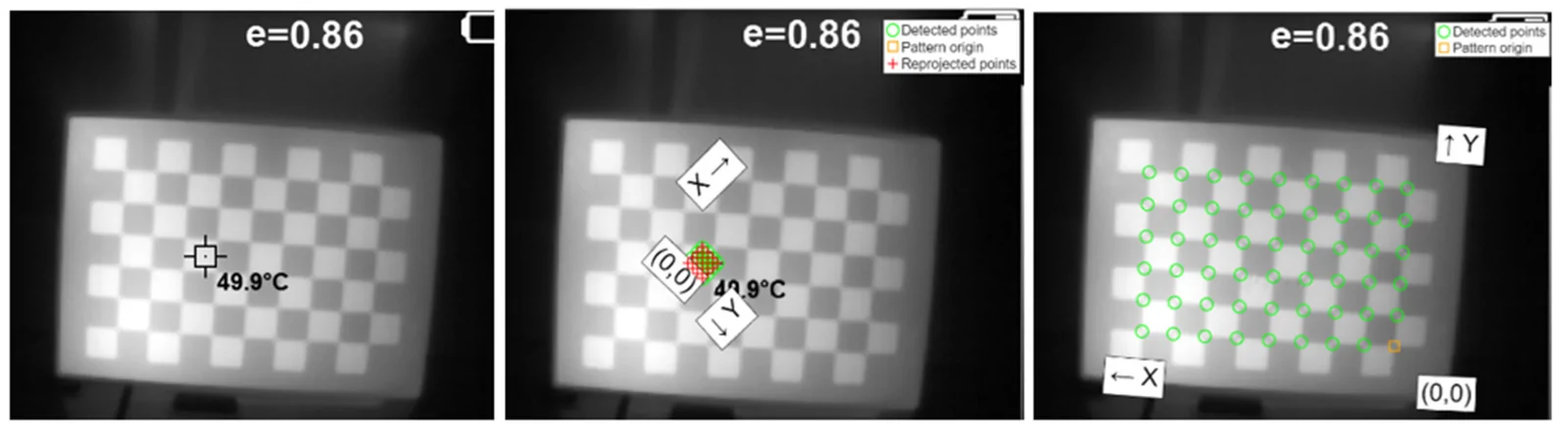
Overlay interference in checkerboard detection. (**Left**): uncorrected image with the firmware cursor over the calibration pattern. (**Middle**): detector output, showing the pattern origin and axes assigned to the cursor rather than the target. (**Right**): the same image after overlay removal, with the complete corner grid recovered.

**Figure 10:**
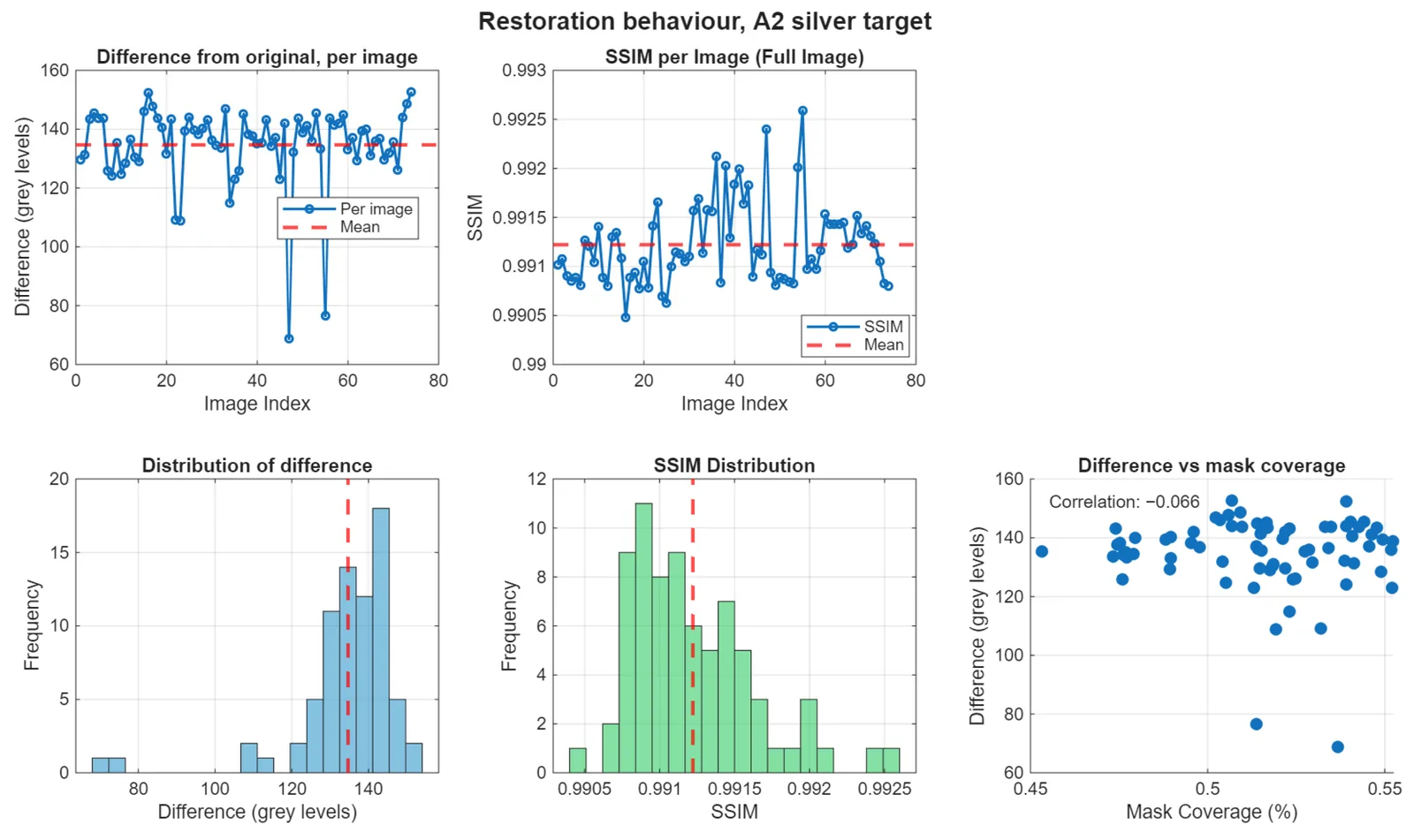

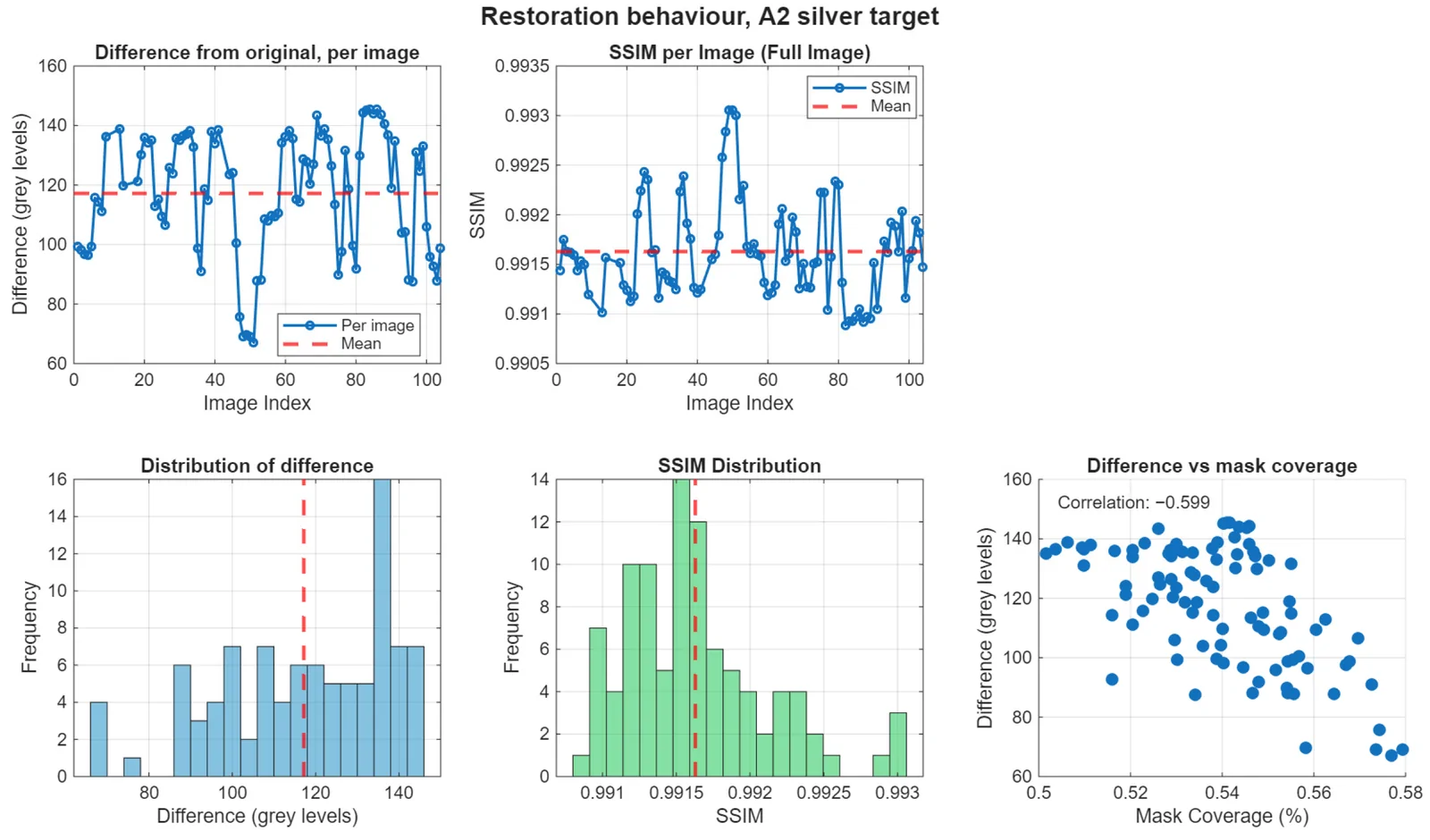
Restoration behaviour for the Silver A2 calibration target under outdoor (**top**) and indoor (**bottom**) conditions.

**Figure 11:**
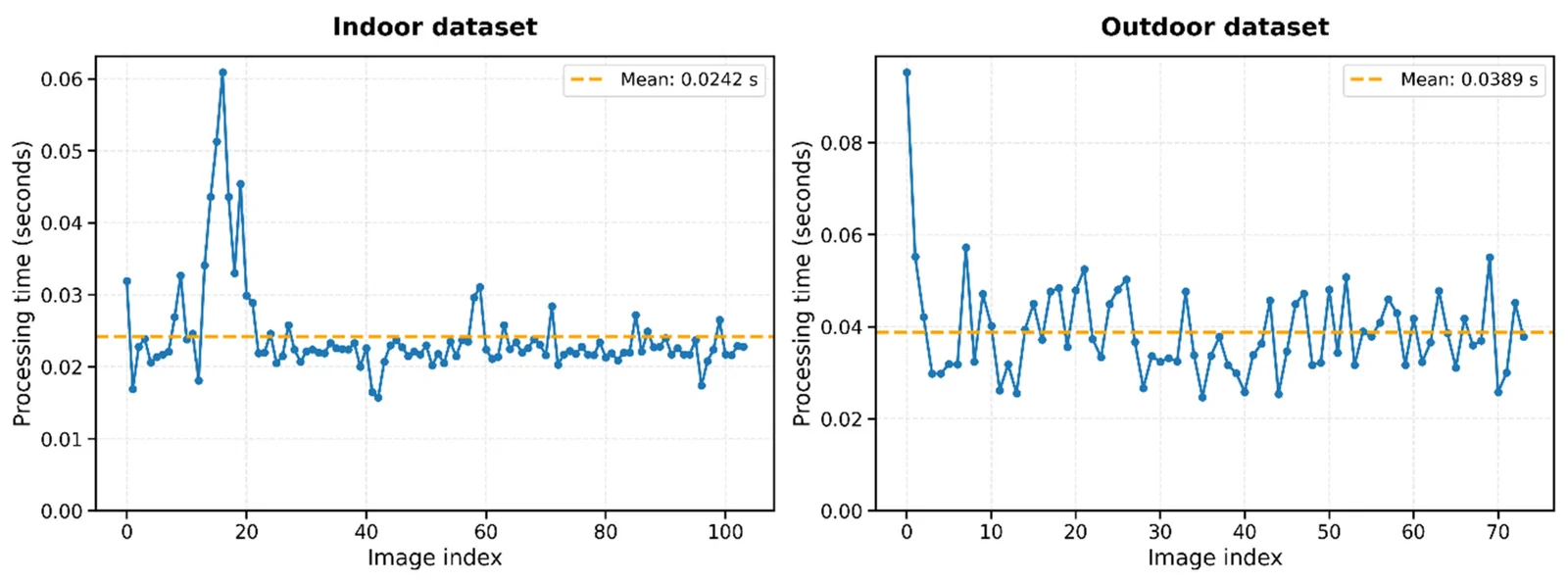
Per-image inpainting processing time for the Silver A2 calibration target under indoor and outdoor conditions.

**Figure 12:**
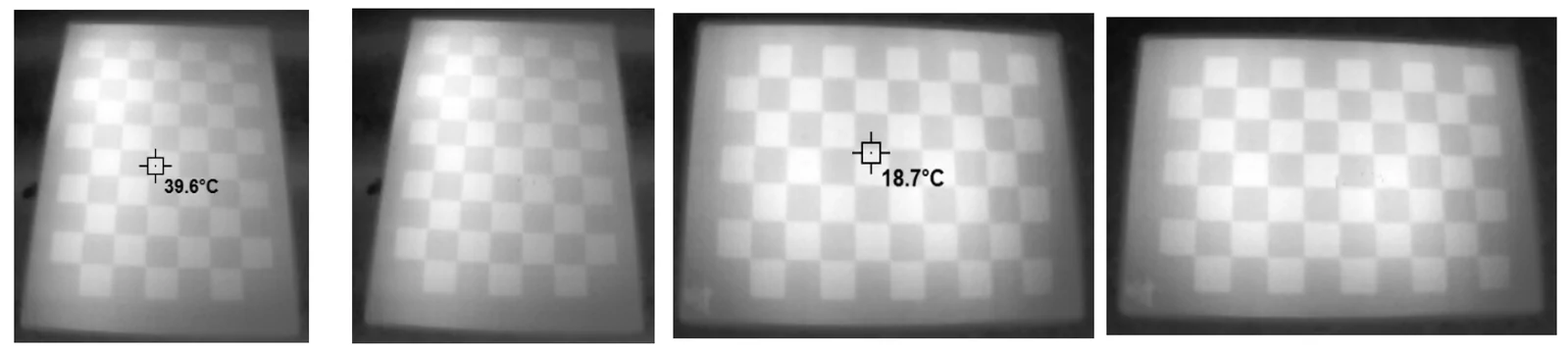
Representative inpainting results for the validation white target.

**Figure 13:**
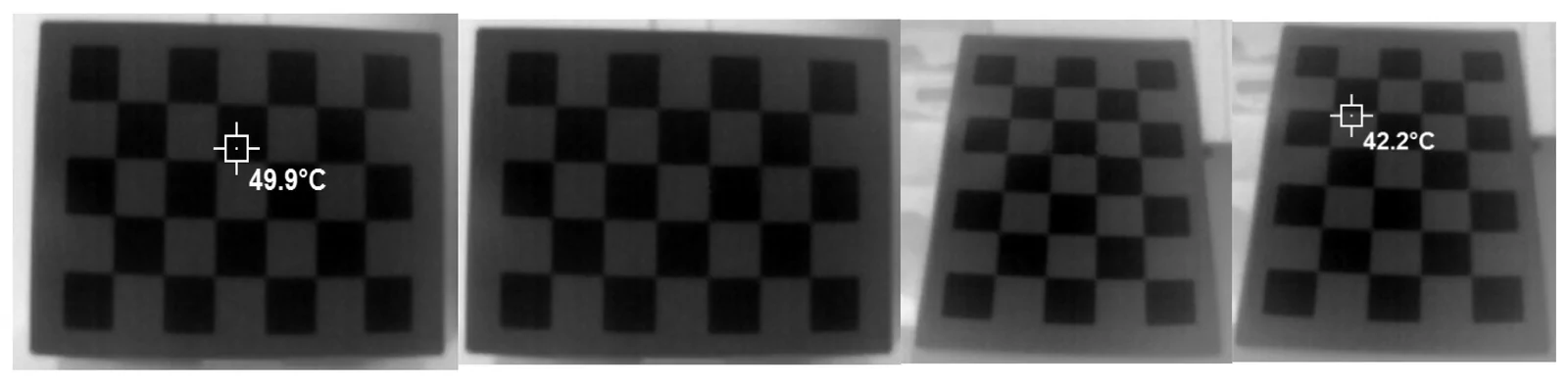
Representative inpainting results for the Silver A3 validation target.

**Figure 14:**
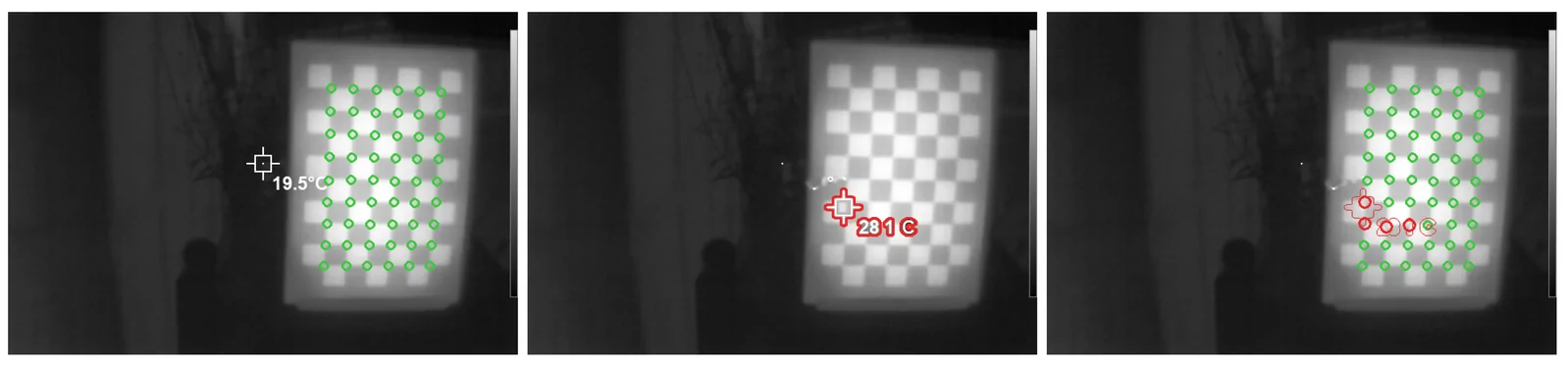
Controlled masking validation. (**Left**): ground truth corner positions in a frame where the firmware overlay falls on the background, leaving the pattern unobstructed. (**Middle**): the extracted overlay composited over the calibration pattern, outlined in red. (**Right**): corners recovered following restoration; those falling within the restored region are marked in red.

**Figure 15:**
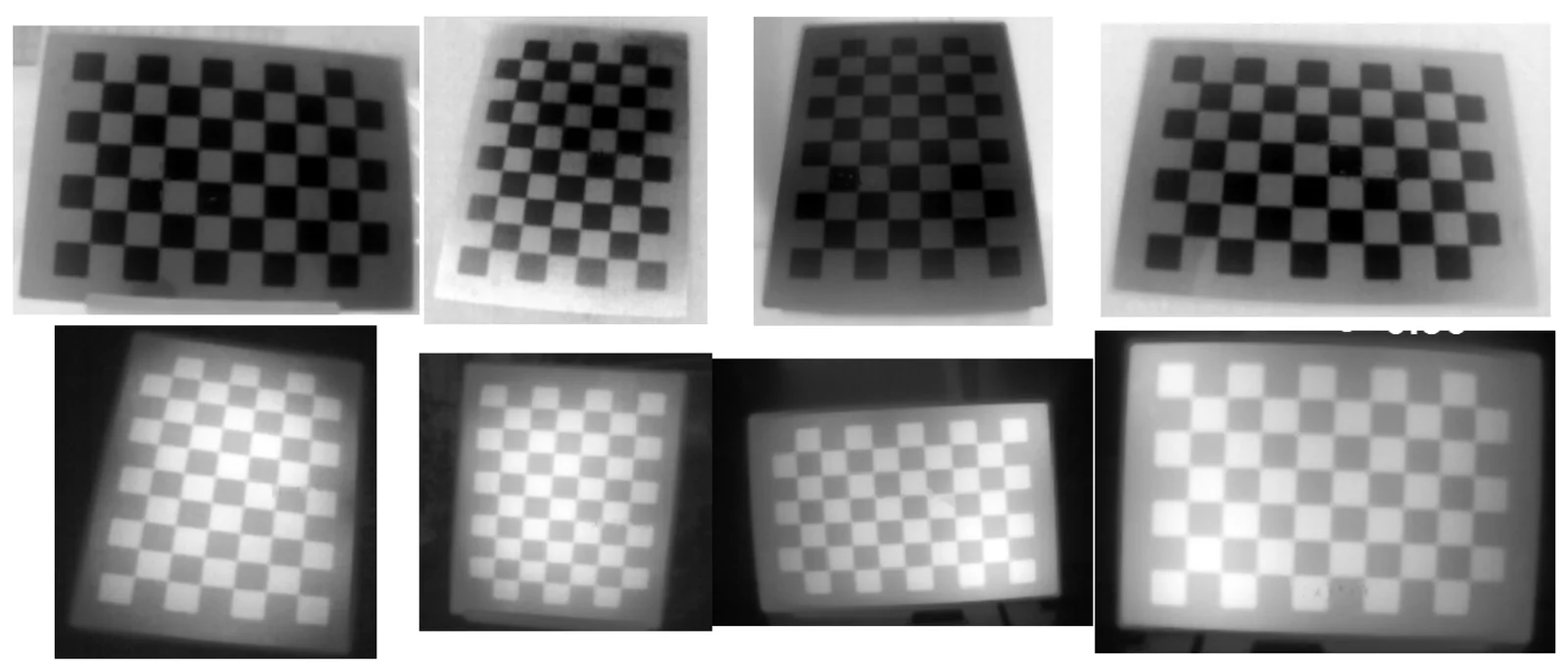
Representative examples of inpainted thermal images for the Silver A2 calibration target under black-hot (**top**) and white-hot (**bottom**) colour palettes.

**Figure 16:**
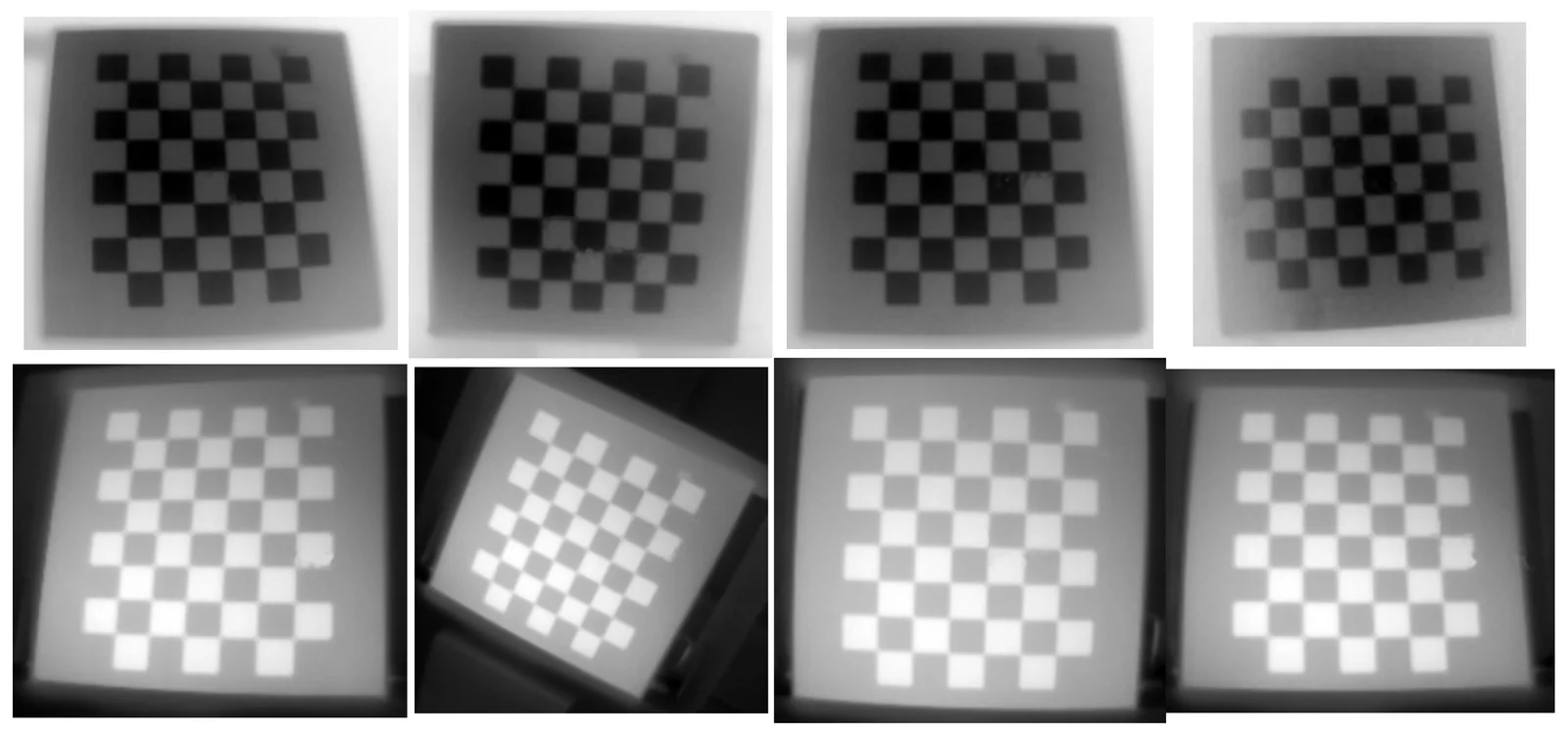
Representative examples of inpainted thermal images for the 230 × 210 mm silver calibration target under black-hot (**top**) and white-hot (**bottom**) colour palettes.

**Figure 17:**
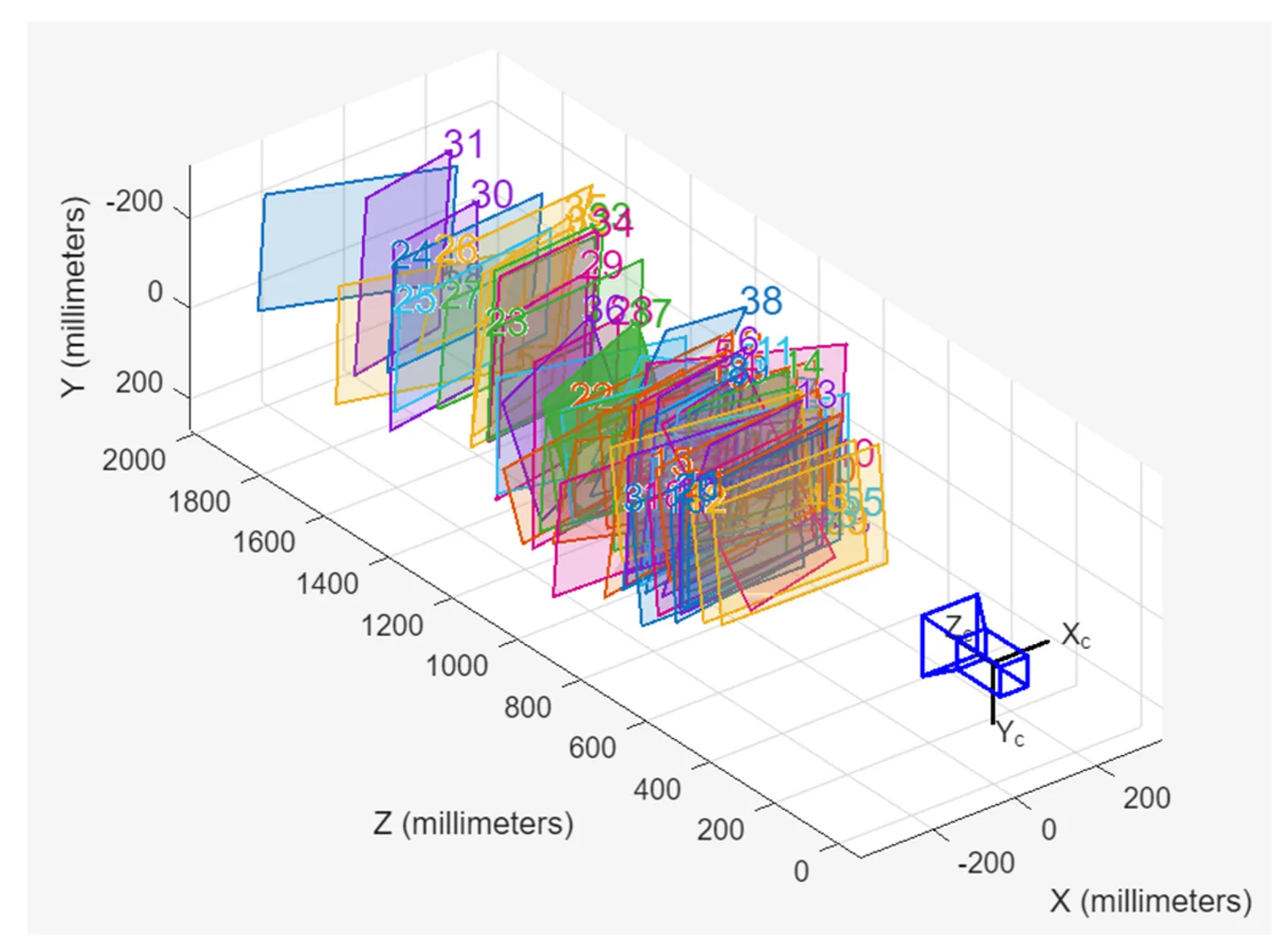
Representative calibration images acquired using the Silver A2 calibration target at working distances of approximately 0.8–2 m.

**Table 1:** Key specifications of the HT-A9 handheld thermal camera used in this study.

Parameter	Specification
IR detector resolution	320 × 240 pixels
Spectral range	8–14 µm (LWIR)
Field of view (FOV)	56° × 42°
Frame rate	9 Hz
Temperature range	−20 to 450 °C
Thermal sensitivity	0.07 °C
Colour palette	Rainbow, iron oxide red, cold colour, black and white, white and black
LCD resolution	640 × 480 pixels
Embedded image overlays	Central cursor and text

**Table 2:** Image export routes available on the HT-A9.

Export Route	File Format	Bit Depth	Radiometric Data	Cursor Present
On-device capture→internal storage	JPG	8-bit	No	Yes
Micro-USB mass-storage transfer	JPG	8-bit	No	Yes
Wireless interface—live stream	—	8-bit	No	Yes
Wireless interface—image download	JPG	8-bit	No	Yes
Manufacturer SDK/API	Not available	—	—	—

**Table 3:** Specifications of calibration target configurations used in this study.

Property	Silver Dibond^®^ (Primary)	White Dibond^®^ (Validation)
Base material	Aluminium composite (Dibond^®^)	Aluminium composite (Dibond^®^)
Panel construction	2× 0.3 mm Al + 2 mm PE core	2× 0.3 mm Al + 2 mm PE core
Panel dimensions	230 × 210 mm; A3 (420 × 297 mm); A2 (594 × 420 mm)	A2 (594 × 420 mm)
Surface	Metallic silver (RAL 9006)	Glossy white
Reverse surface	Slate grey (RAL 7015)	Matte white
Pattern geometry	7 × 8(230 × 210), 7 × 5 (A3); 10 × 7 (A2)	10 × 7
Internal corners	42(6 × 7) 230 × 210; 24 (6 × 4) A3; 54 (9 × 6)	54 (9 × 6)
Square size	25 mm; 50 mm (A3, A2)	50 mm
Printing method	UV flatbed (HP Acuity Press)	UV flatbed (HP Acuity Press)
Ink type	Fujifilm Uvijet KN	Fujifilm Uvijet KN
Role in calibration	Primary calibration and validation	Material independence assessment

**Table 4:** Emissivity measurement of target surfaces. Reference tape ε = 0.95; T_ref = 17.0 °C.

Measurement	T_Tape (°C)	T_Ink (°C)	T_Substrate (°C)	ε_Ink	ε_Substrate
1	50.0	48.0	40.0	0.89	0.66
2	44.0	42.0	37.0	0.88	0.71
3	38.5	35.5	33.0	0.82	0.71
Mean ± SD	—	—	—	0.86 ± 0.04	0.69 ± 0.03

**Table 5:** Sensitivity of the derived emissivities to the assumed reference parameters.

Varied Parameter	Range	ε_Ink	ε_Substrate	Δε
ε ref (T ref = 17.0 °C)	0.93–0.97	0.845–0.881	0.676–0.705	0.169–0.176
T ref (ε ref = 0.95)	15.0–19.0 °C	0.856–0.870	0.670–0.709	0.161–0.186

**Table 6:** Inpainting performance metrics for the primary Silver A2 calibration dataset.

Acquisition Condition	*n*	RMSE	SSIM	Mask Coverage (%)	r
Outdoor	74	135.0 ± 16.2	0.9912 ± 0.0005	0.44–0.56	−0.066
Indoor	104	117.2 ± 20.4	0.9916 ± 0.0005	0.50–0.59	−0.599
Combined	178	124.6 ± 21.2	0.9914 ± 0.0005	0.44–0.59	−0.33

*p* < 0.001 (Pearson correlation between RMSE and mask coverage).

**Table 7:** Computational performance (Silver A2).

Acquisition Condition	Images (*n*)	Mean (s)	Std (s)	Min (s)	Max (s)	Throughput (img/s)
Indoor	104	0.024	0.007	0.016	0.061	41.25
Outdoor	74	0.039	0.010	0.025	0.095	25.73

**Table 8:** Material independence (white vs. Silver A2).

Target	*n*	RMSE	SSIM	Mask Coverage (%)	r
Silver A2	178	124.6 ± 21.2	0.9914 ± 0.0005	0.44–0.59	−0.33
White A2	75	139.4 ± 38.8	0.9911 ± 0.0008	0.31–0.77	−0.540

**Table 9:** Scale validation.

Target	*n*	RMSE	SSIM	Mask Coverage (%)	r
Silver A2	178	124.6 ± 21.2	0.9914 ± 0.0005	0.44–0.59	−0.33
Silver A3	38	151.1 ± 22.0	0.9908 ± 0.0003	0.37–0.97	−0.807
230 × 210 mm	96	117.1 ± 17.9	0.9917 ± 0.0006	0.45–0.55	+0.409

**Table 10:** Comparison of reconstruction methods and inpainting radii.

Method	Displacement, Restored Region (px)	Displacement, Elsewhere (px)	Masked RMSE	Masked SSIM	Time (ms)
No restoration	5.826 ± 3.222	0.045	115.4	0.578	24.4
Mean fill	5.689 ± 3.672	0.056	43.2	0.672	38.6
Median fill	5.612 ± 3.274	0.050	39.9	0.691	32.5
Telea, r = 7	3.537 ± 2.330	0.062	21.4	0.835	44.4
Biharmonic	2.948 ± 2.089	0.044	22.6	0.854	93.7
Navier–Stokes, r = 3	3.996 ± 2.510	0.056	21.5	0.842	38.6
Navier–Stokes, r = 5	3.723 ± 2.500	0.056	21.2	0.843	52.5
Navier–Stokes, r = 7	3.749 ± 2.621	0.058	21.1	0.844	63.9
Navier–Stokes, r = 10	3.802 ± 2.709	0.057	21.0	0.845	40.4
Navier–Stokes, r = 15	3.825 ± 2.551	0.058	20.9	0.845	91.9

**Table 11:** Corner localisation displacement following restoration. There are 192 trials from 16 source frames.

Corner Set	*n*	Mean (px)	SD	95th Percentile	Maximum
Outside restored region	8534	0.048	0.442	0.000	12.33
Within restored region	139	3.338	2.547	7.378	13.47

**Table 12:** Reconstruction metrics computed against true ground truth within the restored region.

Metric	Value
RMSE (grey levels)	21.13 ± 10.57
PSNR (dB)	22.46 ± 3.64
SSIM	0.844 ± 0.068
Whole-frame SSIM	0.989 ± 0.002

**Table 13:** Calibration feasibility.

Configuration	Success	MRE
Without inpainting	0%	N/A
With inpainting	100%	0.155–0.238

N/A: not applicable. Without inpainting, no valid calibration could be obtained, so no reprojection error exists.

**Table 14:** Repeatability of estimated intrinsic parameters.

Parameter	Silver A2 Indoor	Silver A2 Outdoor	Compact Indoor	Compact Outdoor
Images available	61	35	31	32
fx (px)	668.45 ± 3.70 [661.75, 676.23]	668.55 ± 4.73 [660.83, 679.92]	681.79 ± 5.03 [672.36, 691.17]	693.07 ± 7.48 [679.54, 709.88]
fy (px)	667.00 ± 3.91	668.13 ± 4.75	682.72 ± 5.34	693.75 ± 7.62
cx (px)	317.02 ± 1.89	318.02 ± 1.54	318.62 ± 1.82	321.57 ± 1.70
cy (px)	232.87 ± 2.29	230.94 ± 1.15	227.55 ± 1.79	232.07 ± 1.96
k1	−0.4329 ± 0.0088	−0.4413 ± 0.0088	−0.4495 ± 0.0103	−0.4891 ± 0.0160
k2	0.2515 ± 0.0438	0.3123 ± 0.0521	0.3458 ± 0.0785	0.5047 ± 0.0742
MRE (px)	0.191 ± 0.008	0.219 ± 0.005	0.155 ± 0.011	0.238 ± 0.009

**Table 15:** Hold-out validation.

Configuration	Train/Test	Training MRE (px)	Held-Out MRE (px)	Ratio
Silver A2 indoor	43/18	0.188	0.374 ± 0.142	1.99
Silver A2 outdoor	25/10	0.218	0.365 ± 0.136	1.67
Compact indoor	22/9	0.157	0.328 ± 0.286	2.10
Compact outdoor	22/10	0.238	0.538 ± 0.163	2.26

**Table 16:** Comparison of representative thermal camera calibration approaches.

Feature	Zhao & Cao (2024) [17]	Usamentiaga et al. (2017) [11]	ElSheikh et al. (2023) [8]	This Work
Thermal Camera	iTHER-T 3/6	FLIR T450sc	InfraTec VarioCAM HD	HT-A9
Camera Class	Professional	Professional	Professional	Consumer
IR Resolution	640 × 480	320 × 240	640 × 480	320 × 240
Target Size	594 × 420 mm	~220 × 195 mm	150 × 136 mm	594 × 230 mm
Target Material	Paper; circular patterns	Dibond^®^ (Al composite)	Polished SS304 steel	Dibond^®^ (Al composite)
Fabrication	External IR radiation	Industrial UV flatbed	Laser engraving + UV	Commercial UV printing
MRE	0.04% focal length error	0.052–0.233 px (RMSE)	0.057–0.119 px	0.155–0.238 px
Addresses Overlays	No	No	No	Yes
Calibration Success	N/A	N/A	N/A	0%→100%

N/A: not applicable. Calibration success rate is reported only for the present work, as the cited studies used cameras without firmware overlays and therefore did not encounter calibration failure.

## Data Availability

The data presented in this study are available from the corresponding author upon reasonable request. The datasets are not publicly available because they form part of an ongoing PhD research project.

## Abbreviations

The following abbreviations are used in this manuscript:

IRT	Infrared Thermography
LWIR	Long-Wave Infrared
MRE	Mean Reprojection Error
RMSE	Root Mean Square Error
SSIM	Structural Similarity Index Measure
PSNR	Peak Signal-to-Noise Ratio
UV	Ultraviolet
UAV	Unmanned Aerial Vehicle
NS	Navier–Stokes-Based Inpainting
2D	Two-Dimensional
3D	Three-Dimensional
